# When dreams feel real: the MÖBIUS model

**DOI:** 10.1038/s42003-026-09781-x

**Published:** 2026-03-23

**Authors:** Ivana Rosenzweig

**Affiliations:** 1 Sleep and Brain Plasticity Centre, IOPPN, King’s College London, London, UK; 2 Sleep Disorders Centre, Guy’s and St Thomas Hospital, London, UK

**Keywords:** Hippocampus, Sleep disorders

## Abstract

A subset of dreams challenges long-standing distinctions between simulation and memory. These, so-called epic dreams, are defined by immersive realism, emotional neutrality, and persistent autobiographical salience, and can be subjectively indistinguishable from lived experience. They are often recalled with mnemonic authority. In this Perspective, epic dreaming is argued to reflect a systems-level failure of REM sleep’s containment architecture, namely a convergence of neuromodulatory disruption, hippocampal novelty-misclassification, and oscillatory instability that permits internally generated content to enter episodic memory. A probabilistic model of this failure, termed MÖBIUS, is introduced to formalise the conditions under which simulation is mis-encoded as memory.

## Abstract (web summary)

The MÖBIUS model proposes that REM sleep gating failures may mis-tag (pre)replay-like sequences as autobiographical memory, linking MCH-orexin balance, CA2 novelty tagging and θ-γ coupling to dream-reality confusion.

## Introduction

### Dreams, reality, and the fragility of internal simulation

Dreaming has long occupied an ambiguous position between consciousness and memory. Although REM sleep has been consistently linked to vivid, sensorily rich, and emotionally intense dreaming, most dreams are ephemeral, disjointed, and soon forgotten^1,2^. However, a class of dreams stands apart, so-called epic dreams, which violate normative expectations both phenomenologically and cognitively^3–5^. Epic dreaming refers to complaints of “dreaming all night long” together with marked daytime fatigue: patients describe continuous, often trivial or repetitive dream activity and wake feeling as if they had lived an additional working day during sleep^4,6^. Unlike nightmares or lucid dreams, which are anchored by affective or metacognitive extremes, epic dreams need not be dramatic in content; what marks them is their autobiographical weight and the subjective impression of prolonged, coherent lived time^3,7^. In some individuals, including a prototypical case recently reported by our group and comparable patients in our clinical practice, that weight is carried by apparently mundane but highly structured material, extended sequences of everyday events with temporal continuity, stable environments, and often striking affective flatness^8^. Whether fantastical or banal, such dreams can be recalled with unusual confidence and are prone to be erroneously embedded into autobiographical memory, a pattern closely related to what has been termed dream–reality confusion^9,10^. In this Perspective, we use epic dreaming as a clinical lens on a more general problem: under what oscillatory and neuromodulatory conditions can internally generated (pre)replay-like sequences be misclassified as veridical episodic memory? This breach of simulation containment is not merely a curiosity of sleep experience. It reflects a fundamental challenge to our understanding of how the brain segregates imagination from memory.

Clinically, epic dreams have been reported in healthy individuals, but associated dream-reality confusion occurs more frequently in populations exhibiting disrupted REM sleep physiology, namely, individuals with REM sleep behaviour disorder (RBD), narcolepsy, post-traumatic stress disorder (PTSD), insomnia, and other conditions marked by fragmented REM sleep architecture^8,10^. The phenomenon of dream-reality confusion, while not pathological in itself, suggests an underlying failure in the neural mechanisms that normally ensure that internal simulations are sequestered from the systems that construct and maintain belief.

**Fig. 1:**
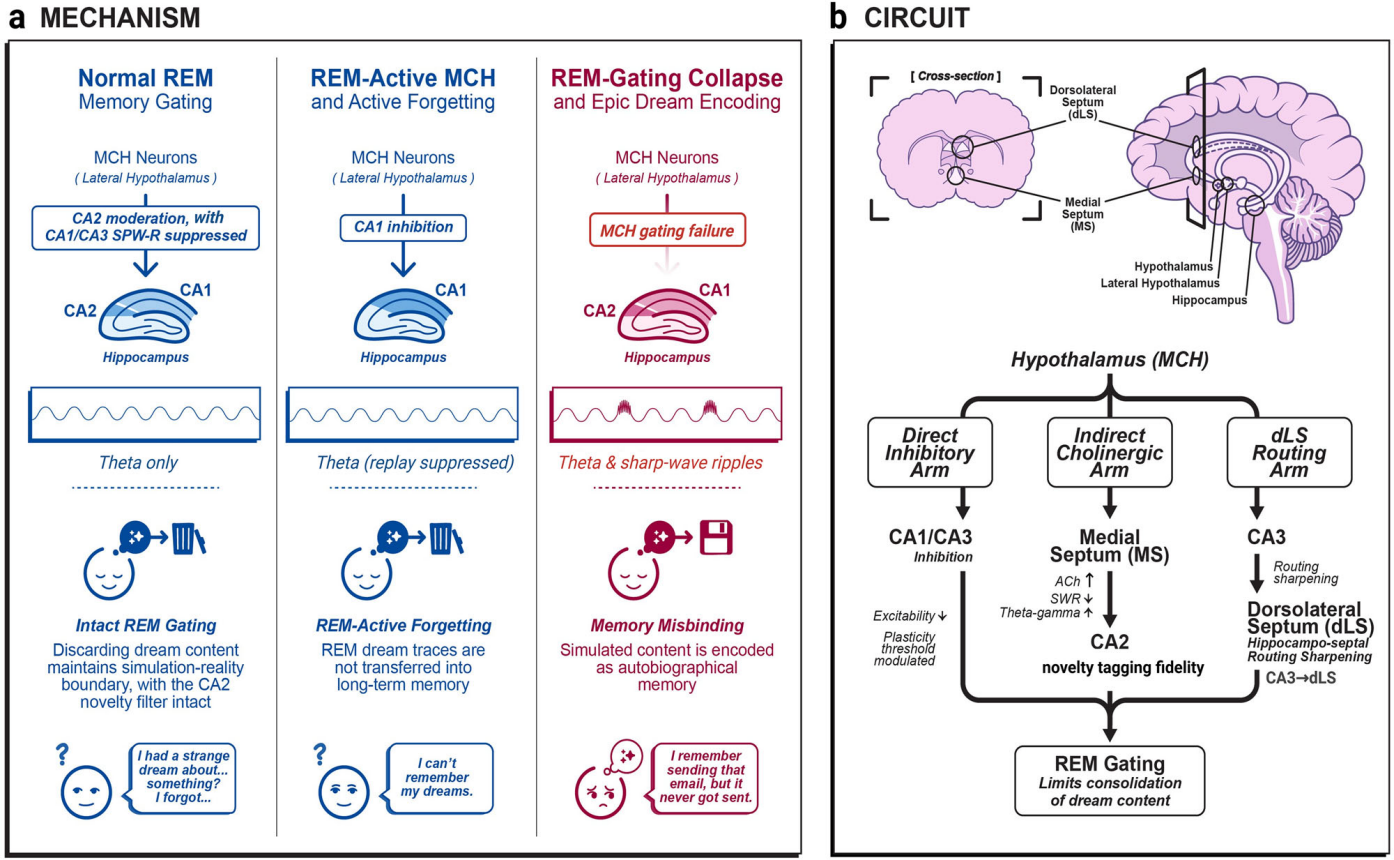
The MÖBIUS model. **a** REM sleep-state transitions and the fate of dream memories. Left: Normal REM sleep memory gating. During physiologically intact REM sleep, MCH neurons in the lateral hypothalamus (LH) moderate hippocampal CA2 activity while CA1/CA3 sharp-wave ripples (SPW-Rs) are suppressed. CA2, a novelty-sensitive subfield involved in contextual discrimination, is kept within a range that prevents internally generated simulations from being tagged as familiar or veridical. Coherent theta oscillations (“theta only”) are preserved, allowing simulation without systems-level encoding. This gating preserves the simulation–reality boundary so that dream content is discarded and typically forgotten; pharmacological enhancement of MCH tone with dual orexin receptor antagonists (DORAs), such as lemborexant, may restore this configuration under conditions of hyperarousal or REM sleep instability. Middle: REM sleep-active MCH and active forgetting. In parallel, REM sleep-active MCH input directly inhibits CA1 pyramidal neurons, raising their plasticity threshold and suppressing SPW-R-mediated replay (“theta, replay suppressed”). Hippocampal reactivation of weak traces is curtailed, biasing REM towards clearance of spurious memory fragments rather than consolidation. As a result, dream traces fail to reach long-term storage and are experienced, if at all, as vague or fragmentary. *Right: REM sleep-gating collapse and epic dream encoding*. When MCH tone is reduced (for example, in stress, post-traumatic stress disorder (PTSD), orexin-dominant states, or pharmacological suppression^17^), CA2 modulation may fail, and internally simulated REM sleep content can be misclassified as familiar or externally grounded. At the same time, SPW-Rs re-emerge in CA1 on a theta background (“theta and sharp-wave ripples”), reinstating consolidation pathways. This conjunction of CA2 misclassification with CA1 replay constitutes a collapse of the REM sleep gate: simulated content is allowed to cross the simulation–reality boundary and to be stored as autobiographical memory. This “memory misbinding” offers a mechanistic account of richly detailed, persistent dream recall and epic-dream–like parasomnia experiences. **b** Circuit scaffold for REM-sleep gating. Lateral hypothalamic MCH neurons project (i) directly to hippocampal CA1/CA3 and (ii) indirectly via medial and dorsolateral septal hubs. The direct inhibitory arm to CA1/CA3 lowers pyramidal excitability and modulates the plasticity threshold, biasing these fields away from ripple-permissive states. The indirect cholinergic arm targets the medial septum (MS), where MCH elevates cholinergic tone; septal ACh, in turn, suppresses SPW-Rs, increases theta–gamma coupling, and, via nicotinic disinhibition in CA2^35^ preserves novelty-tagging fidelity while constraining replay. A third, dorsolateral septum (dLS) routing arm reflects MCH actions in dLS that suppress baseline output yet increase CA3→dLS gain and reduce GABA_*B*_ feed-forward inhibition, sharpening hippocampo-septal routing and further biasing mesoscale dynamics away from ripple-prone configurations. Together, these three arms form an MCH-dependent REM sleep gate that limits the consolidation of dream content and maintains the epistemic boundary between internal simulation and stored experience (see also Supplementary Box 1). dLS dorso-lateral septum, MCH melanin-concentrating hormone, LH lateral hypothalamus, CA1/CA2/CA3 hippocampal subfields, SPW-R sharp-wave ripple, MS medial septum, ACh acetylcholine, REM rapid eye movement sleep. Figure credit: Derek Caetano-Anolles.

In the proposed mechanistic framework, we advance that epic dreaming could result from a coordinated breakdown in three intersecting domains: neuromodulatory gating, hippocampal novelty detection, and oscillatory control of REM microstates (Fig. 1). Specifically, it is argued that a failure of MCH-mediated inhibition, hyperexcitation of hippocampal subfield CA2, and the destabilisation of REM sleep oscillatory manifolds may conspire to render internally generated REM sleep content susceptible to mnemonic consolidation. The ensuing result is not simply a vivid dream, but a memory formed from a simulation, a memory that did not arise from lived experience, but that passes through the same neural machinery and is thus accorded the same epistemic status (Fig. 1).

### The REM architecture of simulation and suppression

REM sleep is paradoxical: cortical activation resembles wakefulness, yet behavioural output is suppressed, and sensory input is attenuated^1^. This paradox is not incidental, but essential to REM’s function as a structured simulation state. In contemporary accounts grounded in predictive coding, REM is conceived as a regime of counterfactual generation^11^. During this phase, the brain, free from external constraints, recombines priors into internally structured sequences that serve to refine hierarchical models of the environment. These simulations support emotional processing, social cognition, and memory reorganisation, among other functions^12–14^. However, these benefits come with a risk. If simulated content is consolidated into autobiographical memory, the system’s internal models are no longer grounded in experience, but in fiction.

To prevent such contamination, REM sleep likely includes multiple active mechanisms designed to prevent encoding. At the systems level, REM is characterised by the suppression of hippocampal sharp-wave ripples (SPW-Rs; predominantly occur during NREM and quiet wake; suppressed by cholinergic tone^15,16^), low hippocampo-cortical coherence, and an oscillatory profile that disfavours consolidation^1^. At the neuromodulatory level, canonical REM sleep is marked by high acetylcholine and low noradrenaline, with serotonin similarly suppressed, a configuration that biases the hippocampus away from externally driven encoding and towards internally generated sequence exploration^11,12^. In clinical epic-dreamers, and in conditions that frequently feature dream–reality confusion such as narcolepsy and PTSD, noradrenergic tone is plausibly elevated relative to this canonical REM sleep baseline, either via trait hyperarousal or stress-related up-regulation of locus-coeruleus output^10,17^. These conditions collectively constrain the transfer of information from the hippocampus to neocortical memory stores. However, this suppression is not uniform, nor is it invulnerable. REM sleep’s architecture is state-sensitive, and when neuromodulatory tone is altered, whether by stress, arousal, pharmacological interference, or sleep disorder, the protective architecture can collapse^1^. Also see Supplementary Box 1.

### Melanin-concentrating hormone and the neuromodulatory firewall

A body of data from preclinical studies suggests that the activity of MCH neurons, located in the lateral hypothalamus and zona incerta, may be central to the gating of REM simulations^18^. Deep‑brain calcium imaging in freely behaving mice confirms that MCH neurons peak in REM sleep, consistent with a role in REM‑specific modulation^19^. In parallel, causal manipulations demonstrate that REM‑active MCH neurons project densely to the hippocampus and promote active forgetting of hippocampus‑dependent memory traces^19,20^. These neurons are preferentially active during REM sleep and project broadly across the brain, including to memory-relevant regions such as the hippocampal CA1 and CA3 subfields, the medial septum, and the prefrontal cortex^21–23^. MCH signalling acts as a regulator of plasticity, long-term potentiation, and a stabiliser of REM architecture^24^. Via septal cholinergic mechanism, MCH-1 receptor activation can bias hippocampo‑septal dynamics away from ripple‑prone states, and toward theta^15,25^. These effects likely collectively reduce the likelihood that internally generated sequences during REM sleep will be treated as salient or consolidated into long-term memory.

At first sight, centring a model of dream–memory boundaries on a peptide may seem unconventional. However, within the current experimental literature, REM-active MCH neurons are uniquely positioned: they fire selectively in REM sleep, project densely to dorsal hippocampus, medial septum and dorsolateral septum, and causal manipulations during REM sleep bidirectionally control retention of hippocampus-dependent memories and septo-hippocampal routing^18–20,24,26,27^. In that sense, MCH already implements the kind of state-locked forgetting signal that epic dreaming appears to lack. Two complementary pathways illustrate the gating architecture mediated by MCH (also see Supplementary Box 1)^28^. First, MCH neurons suppress hippocampal excitability directly, particularly within CA1 and CA3, thereby impeding the CA3–CA1 pathway known to support pattern completion and memory formation. Second, MCH modulates the medial septum, increasing cholinergic tone and thus indirectly regulating theta synchrony across the hippocampal axis^22,23,25^. This synchrony, while essential for simulation, must remain decoupled from consolidation. By regulating both intra-hippocampal excitability (inhibitory) and septal input (excitatory), MCH may act to enforce the boundary between generative activity and mnemonic integration.

Convergent evidence places MCH signalling at the centre of a REM-state ‘firewall’ that limits hippocampo-neocortical broadcast. REM-active MCH neurons densely innervate the dorsal hippocampus; optogenetic activation of their terminals increases GABAergic inhibitory postsynaptic currents (IPSCs) and depresses CA1 pyramidal firing, and closed-loop inhibition of MCH neurons specifically during REM improves retention on hippocampus-dependent tasks, consistent with active forgetting^20,27^. In the septo-hippocampal axis, MCH release in the dorsal lateral septum (dLS) suppresses spontaneous output while increasing CA3→dLS excitatory gain and reducing GABA_B-mediated feed-forward inhibition, thereby sharpening the fidelity of dLS responses to hippocampal input and biasing the mesoscale state away from ripple-prone dynamics^27^. Independently, elevating medial-septal cholinergic tone suppresses sharp-wave ripples and enhances theta^29^. Taken together, reduced MCH tone during REM sleep is therefore a plausible route to ripple resurgence and mis-routing of replay content, our proposed mechanism for dream–reality misbinding under MÖBIUS (Mnemonic Oscillatory Binding of Internally Unverified Sequences), the probabilistic model introduced in this Perspective to formalise when REM simulations become mis-encoded as autobiographical memory.

Disruption of this system, whether via stress-induced orexinergic dominance^17^ or pharmacological MCH antagonism, could compromise the firewall. Under such conditions, simulated content that would normally be ephemeral gains access to encoding pathways. Pharmacological evidence supports this model; dual orexin receptor antagonists (DORAs), which reduce orexin activity and thereby disinhibit MCH neurons, enhance REM sleep continuity and alter dream phenomenology^30,31^. Conversely, impaired MCH signalling is associated with increased REM sleep fragmentation and heightened vulnerability to encoding spurious content^32^. These findings highlight the central role of MCH not merely as a REM stabiliser, but as a neuromodulatory filter that determines whether simulation remains simulation, or becomes memory.

### CA2 as an epistemic filter: novelty, stability, and misclassification

The hippocampus is not a monolithic structure. Within its circuit architecture, individual subfields perform specialised computations that influence the tagging, transformation, and consolidation of experience^33^. Among these, CA2 has recently emerged as a critical site for novelty detection, temporal segmentation, and social salience encoding^34–36^. Despite its narrow width and historical marginalisation, CA2’s distinctive electrophysiological and molecular profile sets it apart from its neighbours^37^. Its pyramidal neurons express dense vasopressin 1b and oxytocin receptors, exhibit resistance to canonical LTP, and are enveloped by robust perineuronal nets, features that collectively confer both computational stability and selective sensitivity^38,39^.

Functionally, converging work in rodents and humans now places CA2 at the intersection of novelty, familiarity, and social salience judgements, rather than simple spatial mapping: its firing reports whether ongoing input accords with previously learned patterns and social contingencies^34,35,40^. On this basis, we treat CA2, heuristically, as an “epistemic filter”: a subfield whose principal contribution is to decide which sequences are tagged as familiar-enough and behaviourally meaningful to be passed to downstream consolidation circuitry, rather than to encode their content per se^41^. This gatekeeping function is even more vital in REM sleep, when the hippocampus is flooded with internally generated sequences. Under normal REM sleep conditions, CA2 likely remains simulated and yet also buffered against overexcitation by MCH-modulated cholinergic tone^35^. Recent studies have demonstrated that CA2 pyramidal neurons occupy a unique computational position: they do not encode experience per se, but rather evaluate novelty and salience by integrating excitatory input from CA3, DG, and entorhinal cortex under tight inhibitory control^28,35,40^.

However, when MCH tone is diminished, CA2 could become ‘vulnerable’. Elevated cholinergic input from the medial septum during REM sleep has been shown to increase CA2 excitability^28,42^, and when this is diminished, this may render its novelty-detection machinery prone to misclassification. Moreover, disruption of this gating, whether through reduced PV+ interneuron activity, increased TREK-mediated leak currents, or stress-driven hypothalamic input, has been recently implicated in social memory errors and dissociative symptoms across neuropsychiatric disorders such as schizophrenia and temporal lobe epilepsy^34,36,43,44^. Whilst still indirect, these findings, thus, arguably also paint a convergent support for the hypothesis that CA2 serves not merely as a hippocampal subfield, but as a precision filter on what is treated as familiar and reliable. In the context of MÖBIUS, we extend this role to the border between internal simulation and encoded memory, acknowledging that this extension remains a mechanistic proposal rather than a demonstrated ability of CA2 to distinguish internal from external events.

Theoretically speaking, in the context of dreaming and sleep, in such a state, simulated REM content that mimics the structural features of experience, coherence, temporal continuity, and goal-directedness could be more easily misidentified as externally valid. CA2 may then erroneously tag the sequence as familiar. This epistemic error would then initiate downstream activation of CA1 encoding circuits and facilitate mnemonic consolidation^28^. Importantly, in this model, the content itself need not be emotionally charged or even interesting; its mundane plausibility may be sufficient to exploit the weakened gate. Thus, as in our patients, the dream would not be remembered because it was extraordinary, but because the systems designed to prevent its encoding failed to recognise it as fictive.

### The REM manifold and oscillatory destabilisation

Thus, REM sleep is not a static state but a dynamic manifold composed of shifting oscillatory microstates^1,45,46^. Within this architecture, coordinated interactions between REM sleep theta–gamma rhythms and the preceding NREM sleep slow-oscillation–spindle dynamics create a high-dimensional temporal scaffold in which simulation unfolds^1,47^. These rhythms are orchestrated by a network of hippocampal interneurons and modulated by medial and lateral septal inputs^27,48^. Parvalbumin-positive interneurons, such as Teevra and Orchid cells, segregate theta phases and direct gamma activity to distinct hippocampal subfields^49–51^. This temporal structuring enables the hippocampus to generate internally coherent sequences without engaging memory consolidation mechanisms^33,48,52^.

Crucially, the integrity of this manifold likely depends on neuromodulatory tone, and possibly particularly that of MCH signalling^18^. When MCH tone is stable, theta-gamma coupling supports the generation of simulation while maintaining containment^53^. However, when MCH is suppressed, whether due to orexinergic interference, stress^17^, or pharmacological dysregulation, oscillatory microstates destabilise^54–56^. Sharp-wave ripple activity may re-emerge inappropriately during REM sleep^25^; theta-gamma coherence may fragment; spindle dynamics may alter^14,57^.

These disruptions open the possibility that internally generated sequences may be synchronised with oscillatory regimes typically associated with memory encoding. Recent work by Yang and colleagues (2024) has demonstrated that hippocampal SPW-Rs can encode not only retrospective traces but also prospective action plans^16^. We treat this awake SPW‑R selection as the empirical anchor for “selection.” Any link from REM sleep preplay to subsequent consolidation is presented here as a working theory to be tested. We suggest that when REM-generated content aligns structurally with such trajectories, for instance, particularly goal-directed, repetitive simulations, it may hijack the same electrophysiological pathways. The consequence in this case would not be a simple heightened dream recall, but mnemonic integration of a simulation.

### Orexin, stress, and the collapse of containment

The functional antagonism between orexin and MCH is a well-established feature of sleep–wake regulation, albeit still far from clearly understood in humans^58^. Nonetheless, according to preclinical and some clinical studies, orexinergic neurons, also located in the lateral hypothalamus, promote arousal and suppress REM sleep initiation and maintenance^20,59,60^. However, we propose that beyond this reciprocal dynamic lies an even more nuanced architecture of neuromodulatory control. Orexinergic microcircuits exert direct inhibitory control over MCH neurons via GABAergic interneurons^58^, and their activity is likely heightened during stress, vigilance, and metabolic drive^17^. Through their dense projections to noradrenergic, histaminergic and other arousal centres, heightened orexin tone can in turn recruit broader monoaminergic systems, further destabilising REM sleep and promoting micro-arousals, so that the MCH gate is challenged from several axes at once^17,58^.

Under pathophysiological conditions, such as insomnia, PTSD, or chronic hyperarousal, it would follow that orexin tone dominates, suppressing MCH activity and destabilising REM sleep transitions^17,18^. This dominance would have cascading effects. For instance, MCH-mediated gating could collapse, CA2 would be rendered dysfunctional, and REM sleep oscillatory architecture would be disrupted. Recent molecular studies have added further complexity. For instance, selective deletion of orexin receptor 2 (Ox2R) in MCH-expressing neurons regulates REM sleep in a sex‑specific manner (female‑biased REM fragmentation; reduced REM theta amplitude)^26^. These findings suggest a hormonally modulated axis of vulnerability and may explain observed sex differences in the prevalence of REM-linked parasomnias and dissociative phenomena in humans^61^.

In such states, the boundary between simulation and memory is eroded not by a single mechanism, but by the convergence of stress-induced neuromodulatory imbalance, hippocampal misclassification, and oscillatory instability. Epic dreaming may thus emerge in our patients not as an exaggeration of normal dreaming, but as a collapse of containment, an epistemic failure shaped by the fragility of REM sleep’s regulatory systems.

### Predictive coding and the misbinding of simulation

From the standpoint of predictive coding, the brain is an inference machine that continually refines hierarchical models of the world to minimise prediction error^11^. REM sleep, devoid of external input, offers a privileged setting for internal generative processes^11,62^. Here, the brain simulates possible futures, recombines priors, and tests model fidelity in a sandbox insulated from sensory perturbation.

However, this sandbox requires constraints. If internal simulations are mistaken for externally validated events, the generative model becomes contaminated. Epic dreaming, in this framework, could be seen as a computational misbinding: a simulated sequence that gains access to consolidation circuits, bypassing metacognitive filters and being encoded as truth. This misbinding is not due to the content itself, but to the failure of the systems responsible for tagging, gating, and filtering internal activity. This aligns with recent findings that suggest that CA2 may act as a conflict detector, not a memory encoder, a gatekeeper of epistemic integrity rather than a spatial or temporal mapmaker. Thus, at the very core of the model is the premise that CA2 may misclassify the (possibly preplayed^63^) sequence, with oscillatory dynamics then permitting its synchronisation with ripple-phase encoding, and with neuromodulatory systems failing to suppress hippocampal plasticity^16^. The brain, unable to distinguish its own fiction, will record the event as memory.

The implications of this model extend beyond dreaming. They speak to broader questions of confabulation, delusion, and the fragile mechanisms by which the brain constructs reality. To illustrate how this theoretical model maps onto clinical reality, we present a structured alignment of symptoms from a recently documented case exhibiting immersive REM sleep dreaming and dream-reality confusion. Supplementary Table S1 summarises the core clinical features and their corresponding neurobiological mechanisms in a previously described case^8^.

**Fig. 2:**
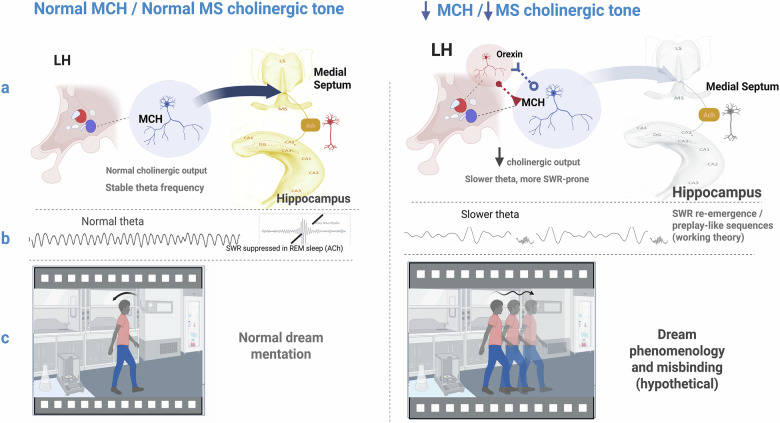
Cholinergic modulation of the medial septum may alter theta rhythmicity and reshape the temporal dynamics of dream content. **a** During REM sleep, REM-active MCH neurons in the lateral hypothalamus (LH) project to the medial septum and hippocampus, supporting medial-septal cholinergic output and stable theta frequency in the hippocampal–entorhinal network (left). Under conditions of reduced MCH tone, with a relative shift toward orexin dominance (right), medial-septal cholinergic drive is diminished, biasing the hippocampus toward a slower, more SWR-prone state. Reduced cholinergic activity from medial septal neurons slows theta oscillations in the medial entorhinal cortex without disrupting grid or speed coding^29^. **b** Schematic traces illustrate normal theta with sharp-wave ripples (SPW-Rs) suppressed in REM sleep under high cholinergic tone (left) and slower theta with SWR re-emergence and preplay-like events under reduced cholinergic tone (right). Under reduced MCH input, this selective modulation may alter the temporal rhythm of internally generated sequences while preserving spatial coherence. It may further lead to the resurgence of SPW-Rs and preplay-like sequences^63,64,72^ being misrecognised as familiar; note that selection of experience by SPW-Rs is an awake phenomenon^16^, and the REM-sleep linkage proposed here is a working theory. **c** Hypothetical consequences for phenomenology: in the normal condition, dream mentation is temporally coherent and readily distinguished from waking experience (left). Under reduced MCH/reduced medial-septal cholinergic tone (right), theta slowing and ripple re-emergence may produce dream experiences that maintain realistic environments, but exhibit disrupted temporal flow, repetition, or loop-like pacing, increasing the likelihood that simulated content is misbound into autobiographical memory. Thus, in the MÖBIUS model, neuromodulatory tone tunes the epistemic integrity of internal simulation by shaping its rhythmic encoding architecture. Created in BioRender. Rosenzweig, I. (2026) https://BioRender.com/uhol1do.

**Fig. 3:**
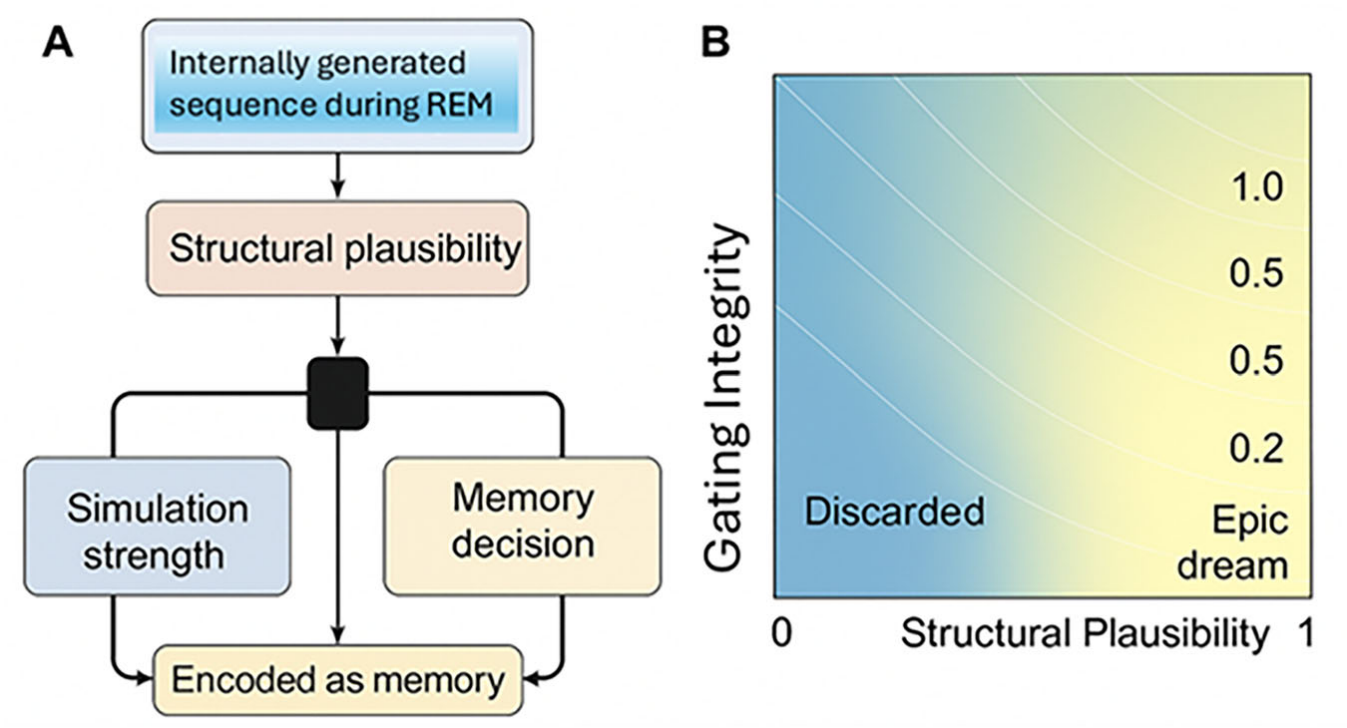
Probabilistic MÖBIUS model of simulation–memory misclassification during REM sleep. **A** Computational flow and decision gate. Internally generated REM sleep sequences *S* are scored for structural plausibility ${{{\mathscr{P}}}}$ (*S*) (e.g., narrative coherence, goal-directedness, temporal continuity). A decision gate integrates ${{{\mathscr{P}}}}$ (*S*) with REM gating integrity *G*(*θ*, *γ*) (a composite capturing septo-hippocampal timing, neuromodulatory tone, and novelty discrimination) to yield the probability that *S* is encoded and later treated as veridical autobiographical memory *M*. Model: P(M = veridical|*S*) =$\sigma (\alpha {{{\mathscr{P}}}}(S)-\beta \,G(\theta ,\gamma ))$, where $\sigma (x)=1/(1+{e}^{-x})$. Higher ${{{\mathscr{P}}}}\left(S\right)$ increases encoding odds; higher $G\left(\theta ,\gamma \right)$ suppresses encoding. **B** Phase-space visualisation of $P\left(M={{{\rm{veridical}}}},|,S\right)$ across ${{{\mathscr{P}}}}\left(S\right)$ (x-axis) and $G\left(\theta ,\gamma \right)$ (y-axis). Contours denote constant probability; the ${{{\rm{high}}}}-{{{\mathscr{P}}}}/{{{\rm{low}}}}-G$ region marks maximal vulnerability to ‘epic dream’ misbinding. The heavy contour indicates $P=0.5$.

Finally, beyond the replay of retrospective traces, recent preclinical work suggests that hippocampal circuits, particularly within CA3 and its projections, may be capable of generating internally structured, prospective trajectories during REM sleep. These sequences, described in rodent models as “preplay”, are not elicited by direct sensory input but emerge from intrinsic dynamics shaped by prior learning and environmental statistics^63,64^. Though their precise status in human REM sleep remains to be elucidated, they appear to instantiate temporally coherent, goal-directed patterns that anticipate future behavioural contingencies^52,65^. We propose, as a working theory, that such preconfigured sequences (‘preplay’^63,64^) become epistemically hazardous when REM sleep containment fails. Selection itself is empirically grounded in awake SPW‑R selection^16^; the REM sleep misbinding step is hypothesised here. In the context of reduced MCH tone or stress-induced neuromodulatory imbalance, these predictive motifs, possibly designed for simulation, may be misclassified as veridical recollection. The resulting memory would not be solely confabulatory in content, but misbound in time (Figs. 2 and 3). For instance, a forward-looking hypothesis encoded as a backward-looking event. This form of temporal misbinding could also arguably offer a neurophysiological substrate for the uncanny realism of epic dreams and may reflect a broader vulnerability in the brain’s mechanisms for segregating internally generated futures from experienced pasts.

To formalise this vulnerability, we propose a probabilistic encoding model that quantifies the likelihood of a REM-generated simulation being misclassified as autobiographical memory (Fig. 3 and Supplementary Box 4). This computational framework, hereafter referred to as the MÖBIUS model (Mnemonic Oscillatory Binding of Internally Unverified Sequences; Figs. 1–3 and Supplementary Fig. S1), captures how the intersection of structural plausibility and REM gating failure can produce persistent dream–memory confusions (Fig. 3; also see Supplementary Boxes 3 and 4 for experimental tests and parameter estimation framework). The model is named after the Möbius strip, a structure with a single surface and boundary, reflecting the collapse between internally generated and externally sourced memory representations (Supplementary Fig. S1). Formally, the MÖBIUS equation is a logistic model with two principled predictors: a structural term describing the dream sequence and a gating term describing the REM sleep physiology in which it occurs. Its novelty lies not in the choice of logistic link, which is standard in systems neuroscience, but in the specific mapping it proposes between measurable neurophysiology, dream phenomenology, and subsequent source memory^66,67^. We aim to capture the balance between the structural plausibility of an internally generated sequence and the neurophysiological integrity of REM gating. Specifically, we define the likelihood that a simulated sequence S will be erroneously encoded as a veridical memory M as follows:

$$P(M={veridical|S})=\sigma (\alpha \cdot {{{\mathscr{P}}}}(S)-\beta {\cdot G}(\theta ,\,\gamma ))$$

The left-hand side, P(M = veridical | S), is the quantity of interest: the probability that a REM-generated sequence S is later stored and treated as a veridical autobiographical memory M. On the right-hand side, ${{{\mathscr{P}}}}$(S) compresses the structure of the dream into a single “plausibility pressure” score, with higher values for sequences that look like ordinary episodes, with continuity, goals and a stable setting. G(θ,γ) does the same for REM sleep gating integrity, summarising theta–theta-gamma organisation, REM sleep continuity, NREM slow-oscillation (SO)–spindle coordination, MCH tone and CA2-mediated novelty control into a scalar where higher values mean stronger containment. The parameters α and β set how strongly each of these composite terms influences the final decision, and the logistic function σ(·) maps their difference onto a probability between 0 and 1. In statistical terms, this is a conventional logistic regression; in mechanistic terms, it states that only when structural pressure outstrips gating integrity should a REM sleep simulation be treated as a memory candidate. The sigmoid function σ(·) reflects the nonlinear transition between simulated content that is appropriately discarded and that which is erroneously admitted into episodic memory.

This formulation encapsulates a core feature of the MÖBIUS model, namely that mnemonic consolidation during REM sleep is not merely a function of content salience, but of containment collapse. Under intact gating, even structurally rich simulations are held in epistemic quarantine. When this containment fails, through septo-hippocampal dysrhythmia, fragmented REM sleep microstates, or attenuated MCH tone, simulations that resemble lived experience in form may cross the threshold into autobiographical memory. Fig. 3 visualises this model. Panel A illustrates the computational architecture: simulated REM sleep content is evaluated along dual axes of structure and gating. Panel B renders the resulting phase space, with encoding likelihood mapped as a function of these variables. In the lower-right quadrant, characterised by high structural plausibility and low gating integrity, simulation is most vulnerable to misbinding. This is the epic dream phenotype: not a failure of imagination, but of epistemic distinction. The sigmoid contour delineates the boundary beyond which internally generated cognition acquires the status of memory.

#### Illustrative empirical analysis

In an exploratory polysomnography (PSG) dataset (4 epic dreamers, 4 matched controls), we operationalised the gating term G(θ,γ) as an amplitude-free composite from central C3–C4: SO–spindle coupling (vector length, logit-transformed), REM sleep continuity (mean bout duration, min), and REM 1/f steepness ( = −slope), each *z*-scored to controls (Supplementary Fig. S2 and Table S2). For orientation, REM 1/f *steepness* is defined as (-)aperiodic slope so that larger values indicate lower cortical excitability (all three features are oriented so larger ⇒ tighter gating). For variance-stabilisation, SO–spindle coupling is logit-transformed prior to control-z-scaling. Epic dreamers showed reduced REM sleep Gating Integrity (RGI_min_; Hedges’ *g* = −1.00), with a concordant separation by signed distance-to-control (*g* = −1.31). A frontal F3–F4 sensitivity analysis yielded a smaller, consistent effect (*g *= −0.71) (Supplementary Fig. S2–S4). Of specific note, given *n* = 4 per group and heterogeneous acquisition, we report Hedges’ g (small-sample corrected) and bootstrap 95% BCa CIs (stratified by group, 20,000 resamples, bias-corrected percentile [BCa]). *p*-values, where reported, use complete randomisation tests enumerating all 70 label permutations for *n* = 4/4. For the primary composite RGI_min_ at C3–C4, the group difference (epic − control) in means was −0.319 with a bootstrap 95% BCa CI [ − 0.628, 0.033]; the corresponding Hedges’ *g* was −1.002 (Supplementary Table S4). Frontal F3–F4 (sensitivity) showed a mean difference of −2.125 with 95% CI [ − 5.578, 0.539] and *g* = −0.706. For the shrinkage covariance, we used Σ_λ_ = (1−*λ*)Σ+*λI* with *λ* = 0.20 (fixed), also see the Supplement. We emphasise descriptive magnitude and direction.; full derivations, control μ/σ, and group values appear in STAR-Methods and Supplement (see Supplementary Tables S2–S4 and Figs. S2–S8).

#### Positioning MÖBIUS within theoretical models of dreaming

The MÖBIUS framework is conceptually aligned with, yet distinct from, several prevailing models of dreaming. Like predictive coding theories, it views REM sleep as a generative state in which hierarchical priors are recombined in the absence of external input. However, whereas these models focus primarily on the constructive functions of dreaming, MÖBIUS introduces the notion of simulation containment, the neuromodulatory and oscillatory safeguards that prevent internally generated sequences from entering episodic memory. In doing so, it augments memory consolidation theories by proposing that REM sleep is not only a filter for salience, but a fragile firewall, whose failure may permit mnemonic encoding of fictive content. Similarly, while affect-based models emphasise the emotional charge of dreams, MÖBIUS highlights structural plausibility and gating collapse as sufficient for persistent encoding, even in emotionally neutral dreams. In this light, the model reframes (epic) dreaming and associated dream-reality confusion not as a heightened variant of ordinary dreaming, but as a systems-level epistemic failure, shaped by the convergence of disrupted REM sleep physiology, impaired novelty discrimination, and oscillatory instability. Rather than opposing existing theories, MÖBIUS complements them by introducing a previously underexamined axis: the fidelity of simulation containment as a determinant of whether dreams are remembered, misclassified, or forgotten (see Table 1). In this respect, MÖBIUS plays the role of a low-dimensional scaffold, analogous to attractor models in working memory or decision-making, designed not to exhaust all mechanisms but to specify which variables matter and which empirical tests should be decisive.

**Table 1:** Comparison of the MÖBIUS framework to existing theories of dreaming

Model	Core focus	MÖBIUS alignment	Novel contribution
Predictive Coding (Friston, Hobson)^11^	Dreaming as internal model testing & refinement	Agrees on REM sleep as a counterfactual generative state	Adds gating mechanisms that prevent misbinding
Memory Consolidation (Stickgold, Born, Diekelmann, Walker)^12,66,67^	Selective REM sleep-based memory integration	Agrees REM sleep usually suppresses consolidation	Introduces failure modes: encoding of simulation
Threat Simulation (Revonsuo)^70^	Dreaming as emotional rehearsal or survival prep	Recognises the function of structured internal content	Highlights that even affect-neutral dreams can persist
Affective Network Models (Nielsen&Levin)^6^	Emotion-driven dysregulation in dream salience	Compatible with stress-induced gating collapse	Shifts focus from emotion to narrative structure & gating
Confabulation/False Memory^71^	Memory errors due to retrieval/encoding distortion	Supports memory misbinding mechanisms	Provides REM sleep-specific, neurobiological substrate (MCH, CA2)

The MÖBIUS model extends, rather than opposes, prevailing theories of dreaming. While most existing frameworks focus on the generation, function, or content of dreams, MÖBIUS foregrounds the containment of simulation and the gating mechanisms that prevent internally generated content from being misclassified as autobiographical memory. The table below provides a structured comparison of how MÖBIUS aligns with, diverges from, and adds to some of the key theoretical models in the field.

### Conclusion: memory without reality

We argue that the concept and phenotype of epic dreaming distinctly provide a window into the machinery of memory construction, belief attribution, and epistemic regulation. These dreams are not merely persistent or vivid; they are misclassified. They reveal that memory is not solely a function of content, nor of emotional salience, but of the physiological context in which information is processed. The hippocampus does not remember what is true; it remembers what passed through the appropriate filters. When those filters collapse, the mind remembers what never was. Those filters are multi-layered, peptidergic (MCH and orexin), monoaminergic (noradrenaline, serotonin), cholinergic and circuit-based, and no single axis will exhaust their behaviour^11,41^. The emerging profile of CA2 dysfunction across psychiatric and neurodegenerative conditions suggests that this subfield may be a keystone in the architecture of reality maintenance, its failure a pivot point for delusion, confabulation, and dream-reality confusion^41,68^.

Understanding the systems that preserve the boundary between simulation and memory, MCH gating, CA2 novelty discrimination, and oscillatory synchrony, offers more than a model of dreaming (Supplementary Box 5). It illuminates how the brain determines what is real. And in doing so, it reminds us that the architecture of consciousness includes not only the ability to generate internal worlds, but the necessity of forgetting them^69^.

See Supplementary Information (separate PDF) for Boxes S1–S5, Fig. S1–S8, and Tables S1–S4, including STAR Methods.

### Outstanding questions

- What are the precise neurophysiological signatures of epic dreaming states, and how do they differ from typical REM sleep?
- How does MCH signalling modulate hippocampal CA2 activity during REM sleep, and what are the consequences of its disruption?
- Can we identify electrophysiological or neurochemical biomarkers that predict susceptibility to persistent dream-reality confusion?
- Does the failure of novelty-tagging in CA2 extend to other forms of internally generated cognition beyond dreaming?
- To what extent does epic dreaming contribute to memory misattribution, false memories, or overgeneralized autobiographical memory?
- Can pharmacological or neuromodulatory interventions (e.g., DORAs, MCH agonists/antagonists, CA2-targeted stimulation) restore REM sleep gating and reduce post-dream intrusion?
- What role do REM sleep microstates and spindle dynamics play in modulating the boundary between simulation and encoding?
- Is there a broader continuum of REM-related cognitive phenotypes that includes parasomnias, dissociation, and certain psychiatric conditions?

## Supplementary information

Supplementary Information

## Data Availability

The datasets generated and analysed during the current study are not publicly available owing to ethical and governance restrictions. Polysomnographic recordings and associated clinical metadata are held within the Guy’s and St Thomas’ NHS Foundation Trust Electronic Research Records Interface (GERRI) under the oversight of the Clinical Research Analytics Governance Group (CRAG) and cannot be transferred outside the Trust firewall. De-identified summary metrics underlying the figures are reported in the main text and Supplementary Tables. Specific, well-founded proposals for secondary analyses under the existing ethical approval may be directed to the CRAG for consideration.

## Code availability

Analysis scripts are archived on Zenodo (DOI: 10.5281/zenodo.18620608)^73^. No participant-level PSG data are included. Control PSG data are available from MASS (O’Reilly et al., 2014); access to the epic-dreaming cohort is governed as described in Data availability.
