# Supplementary material for: When dreams feel real: the MÖBIUS model
Source: Commun Biol. 2026 Mar 23;9:412. doi: 10.1038/s42003-026-09781-x (PMC13009209; doi:10.1038/s42003-026-09781-x)
Supplement: Supplementary file 1 — Supplementary Information [file 42003_2026_9781_MOESM1_ESM.pdf]

Supplementary Materials and Conceptual Integration

Supplementary Box 1. Neurobiological Model of REM Sleep Memory Gating Failure

REM sleep enables the brain to simulate internal models of experience[1]. Under typical conditions, these simulations are prevented from entering episodic memory by a neuromodulatory gating system centered on melanin-concentrating hormone (MCH). This gating system includes two main regulatory arms (and four pathways, please see Table below):

1. Direct modulation of hippocampal Cornu Ammonis 3 (CA3) and CA1 excitability via MCH-1 receptor activation.
2. Indirect modulation of hippocampal theta rhythms and novelty detection through regulation of medial septal cholinergic output, especially affecting CA2.

This dual architecture ensures that simulated REM sleep content is kept separate from mnemonic encoding processes. However, when MCH signaling is impaired, due to stress, elevated orexin tone, or neuromodulatory imbalance, this gating system collapses. REM sleep-generated sequences may then be misclassified as familiar by dysregulated CA2 neurons, and encoded through reactivated sharp-wave ripple (SPW-R) dynamics[2-4]. The hippocampus binds simulated content as if it were autobiographical.

Key elements

- Normal State: REM sleep-active MCH neurons (i) bias CA1/CA3 toward lower excitability and a higher plasticity threshold; (ii) increase medial-septal cholinergic drive to CA2, sustaining novelty-tagging fidelity and indirectly biasing the network away from ripple-prone dynamics; (iii) via the dorsolateral septum (dLS) sharpen hippocampo-septal routing.
- Disrupted State: Reduced MCH tone or orexin dominance → loss of calibrated septal cholinergic modulation at CA2, inappropriate ripple resurgence, and REM sleep instability that degrades novelty-tagging fidelity.
- Outcome: Internally generated REM sleep content can be misclassified as experience and encoded as autobiographical memory.
- Consequence: Dreams gain narrative realism, persistence, and mnemonic weight, hallmarks of the epic-dream phenotype.

Pathways in the MCH gate

Pathway	Mode	Proximal effect	Hypothesised implication for dream gating	Representative refs
MCH → CA1/CA3 (direct)	direct	Lowers CA1 pyramidal excitability; modulates plasticity threshold (MCH input can facilitate LTP in slices); ripple suppression is indirect via cholinergic state	Disfavours encoding of simulated content	Izawa et al., 2019[5] ; Kobayashi et al., 2021[6]; Harris & Burdakov, 2024[7]
MCH → CA1 (REM sleep-timed)	direct (REM sleep)	↑ GABAergic IPSCs during REM; promotes forgetting of weak hippocampal traces	Clears spurious traces	Izawa et al., 2019[5]
MCH → Medial Septum → CA2	indirect cholinergic	↑ Septal ACh → CA2 nicotinic disinhibition → novelty-tagging fidelity; ACh suppresses SPW-Rs	Maintains novelty filter; contains replay	Vandecasteele et al., 2014[2]; Lu et al., 2013[8]; Zhang et al., 2021[3]; Pimpinella et al., 2021[9]
MCH → dorsolateral septum (dLS)	indirect via dLS	MCH in dLS suppresses baseline output while increasing CA3→dLS excitatory gain and reducing GABA_B feedforward inhibition; biases mesoscale state away from ripple-prone dynamics	Sharpens hippocampo-septal routing; reduces broadcast of simulated content	Liu et al., 2022[10]

Abbreviations: ACh, acetylcholine; dLS, dorsolateral septum; IPSC, inhibitory postsynaptic current; MS, medial septum; SPW-R, sharp-wave ripple.

Notes

- Ripple suppression during REM sleep is framed as an **indirect consequence of elevated cholinergic state** rather than a direct MCH action within CA fields.
- Evidence strength: CA1 direct effects are best supported; CA3 involvement is anatomically plausible (MCHR1) but functionally less established. The dLS pathway

is emerging and mechanistically consistent with hippocampus–septal routing effects reported in vivo.
- MCH has been shown to lower the plasticity threshold (weak-LTP facilitation; metaplasticity) in one study[7], but it enforces REM sleep -state containment (reduced CA1 excitability; septal–CA2 control). Which facet dominates depends on subfield and brain state.

This mechanistic scaffold offers the hypothetical neurobiological foundation for understanding how REM simulation can breach its containment boundary and be mistaken for lived memory. Within this framework, Buzsáki's "preconfigured brain" hypothesis offers a computational complement[11, 12]: the hippocampus generates internally structured neuronal sequences even in the absence of external input. In this Perspective, awake SPW-R selection is the empirical selection anchor[13]; REM sleep preplay → misbinding remains a testable working theory. These intrinsic trajectories, normally shaped and grounded by sensory experience, may, under disrupted REM sleep conditions, be activated and bound to dream content. When the usual gating mechanisms fail, these sequences are no longer dismissed as fictive or novel. Instead, they are treated as valid input, resulting in the misclassification of simulation as autobiographical memory. Epic dreaming, thus, reveals the vulnerable interface between preconfigured internal dynamics and memory systems built for adaptive plausibility, not epistemic certainty.

Supplementary Box 2. Phenomenological Markers of Epic Dreaming

Epic Dreaming in Practice: Structural Markers from a Clinical Case

The following are illustrative dream elements recorded by a patient with REM sleep without atonia, persistent dream-reality confusion, and immersive, emotionally neutral dreaming[14]. These excerpts highlight recurring features that define the "epic dreaming" phenotype. Each supports the proposed model of REM sleep containment failure and memory misbinding.

• Narrative Realism: "I dreamt a full workday, complete with AV issues, a colleague named X, and feeling judged for eating habits, details indistinguishable from waking life."
• Spatial Navigation: "I navigated between unfamiliar train stations with clear goals and transitions, making travel decisions with internal logic."
• Nested Cognition: "I dreamed I was relaying the dream to my therapist. This recursion didn't trigger lucidity, only more immersion."
• False Sensory Memory: "In the dream, I tasted a crumble, then realized it was a disgusting ham and egg bake. That flavor memory still lingers."
• Post-Sleep Confusion: "I checked Slack and email in the morning to confirm conversations hadn't really happened."

Supplementary Box 3. Experimental Testing of the REM Sleep Containment Model

We propose a set of cross-species experimental paradigms to test the MÖBIUS model. These experiments aim to test the model's core prediction, namely that simulated REM sleep sequences are misclassified and encoded as memory when structural plausibility is high and gating mechanisms are compromised.

I. Rodent Models: Circuit Causality

1. Closed-loop REM sleep gating modulation
• Setup: Use optogenetic or chemogenetic manipulation in rodents implanted with hippocampal tetrodes. Closed-loop detection of SPW-Rs or theta-gamma desynchronisation during REM sleep.
• Manipulations: (a) Activate MCH neurons to suppress memory encoding; (b) Inhibit CA2 pyramidal neurons.
• Readout: Post-REM sleep memory performance, REM sleep ripple reactivation, theta coherence.
• Hypothesis: Suppressing ripple re-entry or CA2 activity will block dream content consolidation.

2. Preplay misbinding paradigm
• Task: Allow animals to explore novel environments that structurally resemble simulated sequences previously observed during REM sleep.
• Manipulation: Disrupt MCH gating during REM sleep using pharmacological antagonism.
• Readout: Increased behavioral recall of simulated (unexperienced) sequences.
• Hypothesis: Preconfigured hippocampal trajectories will be encoded as real memory under disrupted gating.

II. Human Neuromodulation and Cognitive Testing

1. Non-invasive hippocampal modulation during REM sleep
• Techniques: Transcranial focused ultrasound (tFUS) or Temporal Interference (TI) stimulation.
• Targets: CA2 or medial septum.
• Protocol: Apply stimulation during PSG-confirmed REM sleep.
• Readout: EEG markers (theta-gamma coherence, REM sleep microstates), dream reports, post-sleep source memory accuracy.
• Hypothesis: Preserving gating integrity via stimulation will reduce memory misbinding of dream content.

2. Dream-source misattribution paradigm
• Task: Participants record REM sleep dream reports across a week.

- Procedure: At follow-up, present mixed real and dream content in a source memory test.
- Analysis: Correlate dream misattribution rates with REM sleep architecture and neurophysiological metrics.
- Hypothesis: Misattributions will scale with structural plausibility and disrupted REM sleep gating features.

Supplementary Box 4. Estimating Parameters α and β in the MÖBIUS Model

This box outlines a testable experimental framework for estimating the two key parameters of the MÖBIUS model: α, which captures the structural plausibility of REM sleep dream content, and β, which reflects the integrity of REM sleep gating mechanisms. Using a combination of dream report scoring, polysomnographic recording, EEG-based coherence analysis, and post-sleep source memory testing, these parameters can be empirically approximated and fit using the proposed probabilistic model.

Estimating α (Structural Pressure)

α can be estimated by scoring dream reports on several dimensions:
- Narrative coherence (story structure, logical flow)
- Temporal continuity (chronological progression of events)
- Goal-directedness (presence of intention or planning)
- Vividness and sensory detail (perceived realism)

These metrics can be aggregated into a composite plausibility score, $\mathcal{P}(S)$, which is then regressed against the rate of dream–reality confusion in post-sleep source memory tests.

Estimating β (REM Gating Fidelity)

β can be estimated using neurophysiological measures of REM sleep stability and gating integrity:
- Theta-gamma coupling (EEG coherence during REM sleep)
- REM fragmentation (number and stability of REM sleep bouts)
- Oscillatory microstate stability (transitions between REM sleep substates)
- MCH-related activity (inferred pharmacologically or via indirect biomarkers)

Together, these markers define G(θ, γ), the REM sleep gating function. Higher values of G suppress the likelihood of encoding, allowing for empirical estimation of β.

Experimental Design Overview

An experimental design to estimate α and β could include the following steps:
1. Collect multi-night dream reports from participants during PSG-monitored sleep.
2. Score dreams for structural plausibility.
3. Measure REM sleep neurophysiological markers (EEG, microstates, gating indicators).
4. Conduct a source memory test comparing dream content to waking experience.
5. Fit the MÖBIUS equation using logistic regression:

$$P(M = \text{veridical} \mid S) = \sigma(\alpha \cdot \mathcal{P}(S) - \beta \cdot G(\theta, \gamma))$$

Axes and scaling: For visualizations such as Fig. 3B, $\mathcal{P}(S)$and $G(\theta,\gamma)$ should be placed on a common scale. In empirical applications we recommend control-referenced z-

scoring for each component feature and for the resulting composite(s), which yields unit-free weights α and β and makes effect sizes interpretable across cohorts.

Operational proxy for $G(\theta, \gamma)$**:** In our PSG illustration, $G(\theta, \gamma)$is operationalized using an amplitude-free REM Gating Integrity composite ($\mathrm{RGI}_{\mathrm{min}}$): the equal-weight mean of control-normalized z-scores for SO–spindle coupling (logit r), REM bout continuity (mean bout duration), and REM 1/f steepness (defined as $-$aperiodic slope; larger values indicate lower excitability). Larger $\mathrm{RGI}_{\mathrm{min}}$ indicates tighter REM containment. Full definitions and sensitivity analyses are provided in the Supplementary Methods and Supplementary Figures/Tables.

Worked example (toy values): If $\mathcal{P}(S) = +1\mathrm{SD}$, $G(\theta, \gamma) = -1\mathrm{SD}$, and $\alpha = \beta = 1$, then $\sigma(\alpha\mathcal{P} - \beta G) = \sigma(2) \approx 0.88$, indicating high vulnerability to misbinding under high structural plausibility combined with reduced gating integrity.

Cross-references: See Supplementary Figures S2–S8 and Tables S2–S4 for computation of $\mathrm{RGI}_{\mathrm{min}}$, bootstrap CIs, and sensitivity analyses.

Supplementary Box 5. Epistemic Containment Beyond REM Sleep: Toward a Systems-Level Account of Memory-Reality Boundary Failure

The MÖBIUS model was developed to describe a failure of REM sleep's containment architecture, a state in which internally generated simulations, typically sequestered from mnemonic consolidation, are erroneously tagged and stored as autobiographical memory. However, converging evidence suggests that the model's core vulnerability, namely, a breakdown in systems that discriminate simulation from experience, may extend beyond REM sleep. Here, we outline how the same neurophysiological mechanisms implicated in epic dreaming may also underlie waking phenomena such as déjà vu, dissociation, and false memory formation.

At the center of this generalizable framework lies the hippocampal subfield CA2, whose role in novelty discrimination, social familiarity tagging, and contextual segmentation has recently been elucidated. In both rodent and human studies, CA2 exhibits a unique resistance to plasticity, strong perineuronal net stabilization, and dense modulatory input from the medial septum and hypothalamus. These features make it a candidate epistemic filter, a region specialized not for encoding experience, but for determining what should be encoded.

Under normal conditions, REM sleep prevents the consolidation of dream content through MCH-mediated inhibition, suppression of sharp-wave ripples (SPW-Rs), and decoupling of hippocampo-cortical circuits. However, when this architecture collapses, whether due to stress, REM sleep fragmentation, or neuromodulatory imbalance, simulated sequences gain access to consolidation pathways. This produces the epic dream phenotype: mundane yet persistent, structurally coherent yet fictive memories.

Remarkably, this pattern mirrors features of déjà vu/vecu and dissociative states, both of which are characterized by:
- A sense of false familiarity (CA2 mistagging),
- Emotional flattening or detachment (reduced affective salience),
- Confusion between imagined and experienced events (source memory failure),
- And co-occurrence with conditions involving REM sleep disruption, hippocampal dysrhythmia, or neuromodulatory dysregulation [e.g. post-traumatic stress disorder (PTSD), temporal lobe epilepsy, narcolepsy].

The concept of hippocampal "preplay", preconfigured, internally generated trajectories, offers further explanatory power. Studies have demonstrated that hippocampal circuits can produce goal-directed, temporally coherent sequences in the absence of external input, shaped by prior learning[15]. When these preplay events are misclassified as veridical, they

may become the neural basis of false memory or subjective déjà vécu. This form of temporal misbinding, where the future is encoded as past, parallels the failure mode described in MÖBIUS for REM sleep-based simulation encoding.

We do not claim equivalence between dreaming and dissociation, nor between REM sleep misbinding and waking confabulation. However, we propose that these states may reflect distinct manifestations of a common systems-level failure: the collapse of internal containment, the breach of an epistemic boundary that normally ensures internally generated sequences are quarantined from belief.

Thus, MÖBIUS may serve not only as a model of REM sleep dreaming that leads to wakeful false memories and dream-reality confusion, but as a template for understanding how the brain adjudicates epistemic status, how it determines what is real, what is imagined, and what must be forgotten.

Supplementary Table S1.A. Mapping Clinical Features to Theoretical Model of REM Sleep Gating Failure

Clinical Feature	Observed in Case[14]	Mapped Theoretical Mechanism
REM Sleep Without Atonia (RSWA)	Confirmed by video-polysomnography; dream enactment recorded.	Breakdown of REM sleep containment; instability in REM sleep gating architecture.
Persistent Dream–Reality Confusion	Frequent reality-checking behaviors post-waking; confusion about real vs. dreamed events.	Failure of novelty discrimination in CA2; misbinding of simulated content as memory.
Immersive, Narrative-Coherent Dreaming	Reported dreams as mundane, continuous, and structured like real experiences.	REM sleep manifold integrity collapse; theta–gamma disruption allows mnemonic encoding.
Emotionally Neutral, Yet Vivid Dreams	Dreams lacked emotional salience but were remembered with high fidelity.	Low affective tagging bypasses usual metacognitive filters.
Cognitive Fatigue After Sleep	Described sleep as mentally exhausting; ‘second shift’ phenomenon.	Continuous simulation without gating; sustained hippocampal activation.
Partial Response to Vortioxetine	Improvement in dream–reality confusion but persistence of immersive dreams[14]	Consistent with possible serotonergic modulation of prefrontal reality-monitoring circuits.

The key clinical features observed in the reported case[14] map onto specific components of the proposed theoretical model of REM sleep containment failure. Each symptom is linked to a mechanistic hypothesis, bridging the phenomenology of epic dreaming with underlying neurobiological processes. The case thereby serves as a natural probe into the proposed breakdown of simulation-memory boundaries during REM sleep.

Supplementary Table S1.B. Dream Vignettes and Phenomenological Markers[14]

Theme	Dream Excerpt	Phenomenological Marker
Narrative Realism	I dreamt a full workday, complete with AV issues, a colleague named X, and feeling judged for eating	Autobiographical embedding, social context realism.

	habits, details indistinguishable from waking life.	
Spatial Coherence & Navigation	I navigated between unfamiliar train stations with internal logic about which train I needed, and spoke to strangers as if familiar colleagues.	Hippocampal spatial mapping, navigational fluency.
Nested Metacognition	I dreamed I was telling my therapist about the dream I'd just had. It felt recursive but coherent.	Dream-within-a-dream structure; metacognitive leakage.
Sensory Misbinding	I tasted apple crumble in a dream. It turned into a savoury baked egg dish that left a vivid taste memory, despite never existing.	False episodic memory; sensory realism.
Reality-Testing Behavior	I woke up and checked emails and calendars to verify whether certain conversations had really happened.	Post-dream confusion; compensatory reality monitoring.

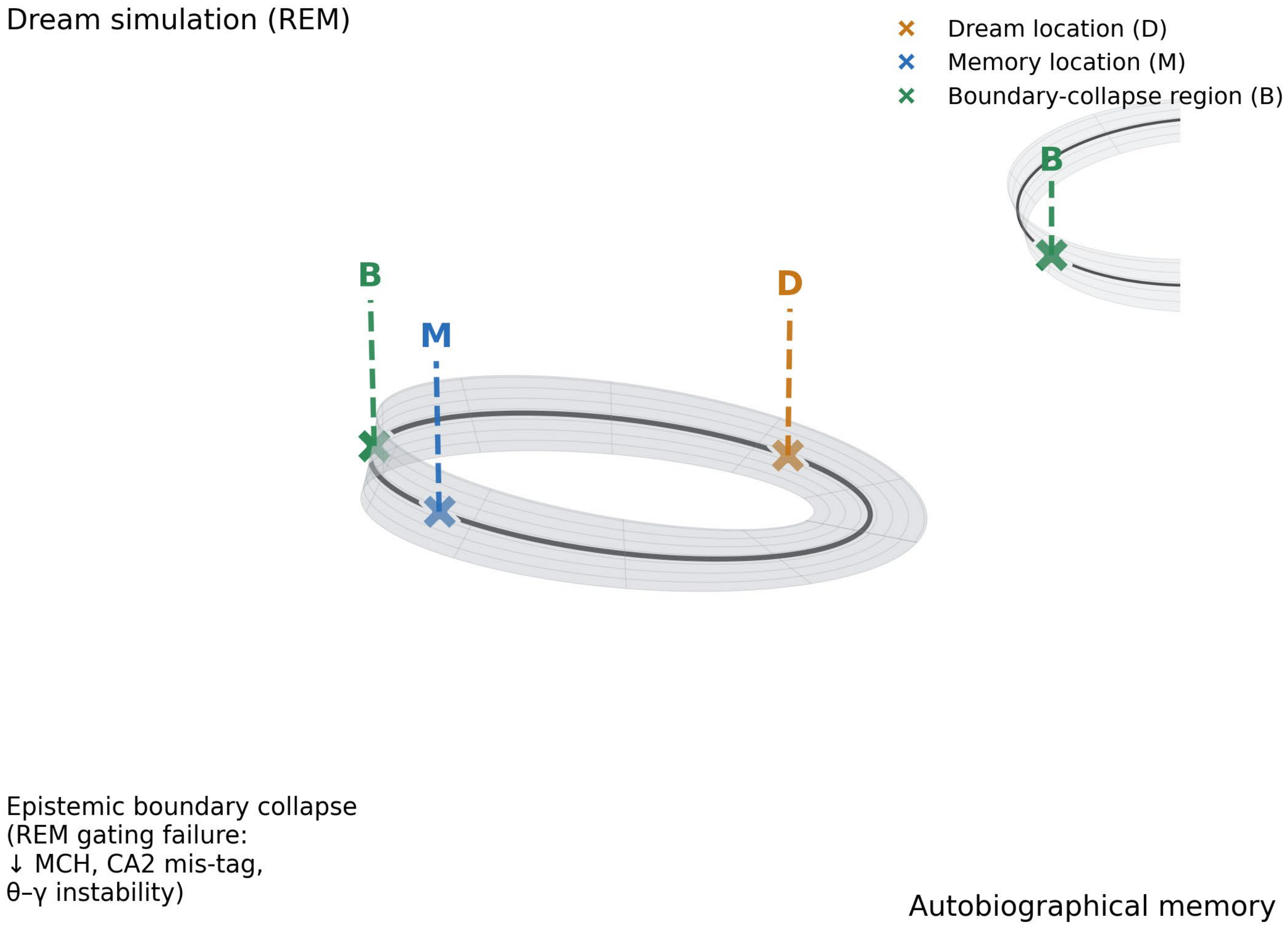

Supplementary Figure S1. The MÖBIUS manifold and the boundary-collapse locus for dream–memory misbinding.

A parametrically gridded Möbius strip illustrates the core hypothesis of the MÖBIUS model (Mnemonic Oscillatory Binding of Internally Unverified Sequences). This schematic Möbius strip representation is used to formalise how internally generated REM sleep simulations can, under specific failures of state-dependent gating, be misclassified as autobiographical memory. The grey Möbius surface depicts a single continuous representational manifold; the black midline indicates a representative mnemonic trajectory along the one-sided surface. The orange marker (D) denotes a dream-simulation location during REM sleep. The blue marker (M) denotes the corresponding autobiographical-memory locus on the same manifold. The green marker (B) highlights the boundary-collapse region (magnified inset), where REM sleep gating failure, hypothesised to involve reduced melanin-concentrating hormone (MCH) tone, CA2 mis-tagging, and/or theta–gamma instability, permits REM sleep-derived simulations to be encoded with autobiographical authority.

Mathematical Formulation of the REM sleep Gating Integrity (RGI) Index and Concise Methods

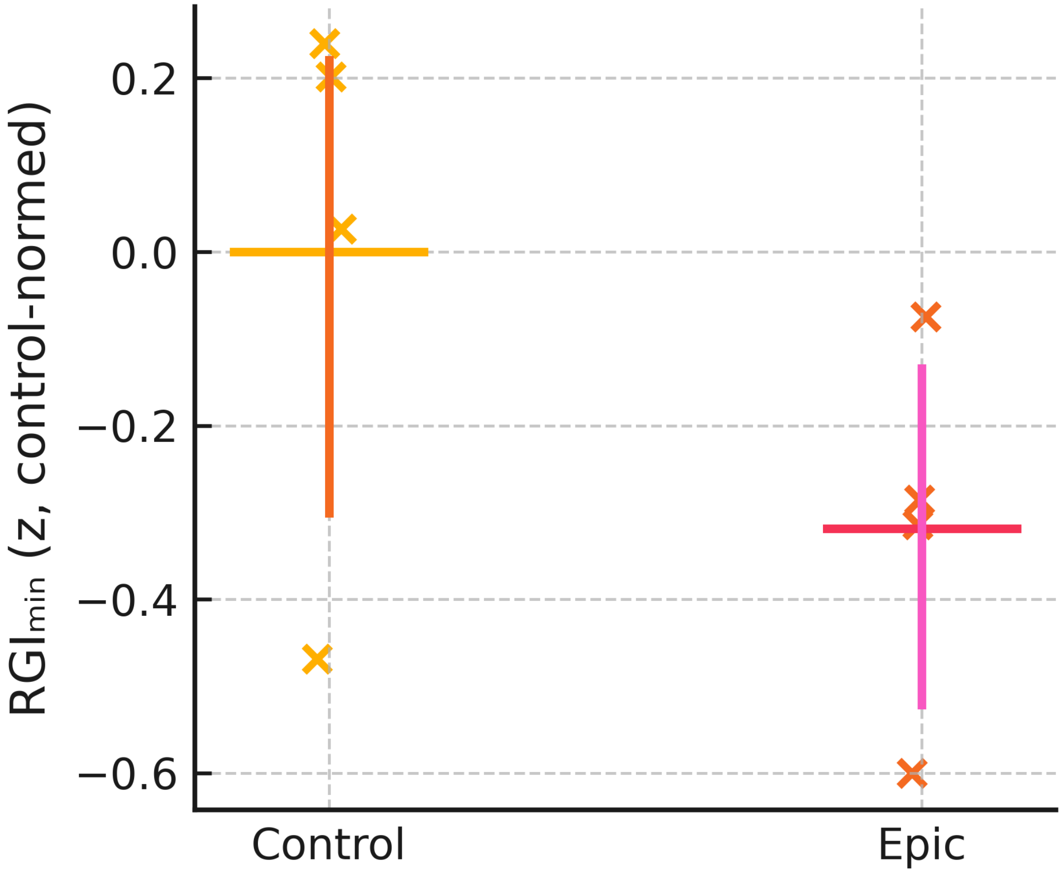

Supplementary Figure S2. REM Sleep Gating Integrity (RGI_{min}), C3–C4: epic dreamers versus controls.

Jittered points demonstrate values for controls and epic dreamers, with group means (horizontal bars) and bootstrap 95% confidence intervals (BCa; vertical whiskers; 20,000 resamples) also shown. Point colour encodes group identity only (Control vs Epic); it carries no quantitative meaning. The composite RGI_{min} is the equal-weight mean of control-normalised z-scores for SO–spindle coupling (vector length, logit-transformed), REM sleep continuity (mean bout duration, minutes), and REM sleep 1/f steepness (= −slope; larger = steeper = lower excitability). Larger values indicate tighter REM sleep containment (see Supplementary Methods). Effect size for this dataset: Hedges' g = −1.002. RGI is control-z-scaled and amplitude-free (logit-transformed SO–spindle coupling, REM sleep continuity in minutes, REM 1/f steepness = −slope). Supplementary

Figures S3–S8 below expand the analyses summarized here. Abbreviations: RGI, REM Gating Integrity; SO, slow oscillation; CI, confidence interval.

See Supplementary Figures S2–S8 for distributions, weights and sensitivity analyses: S2 (RGI_min), S3 (weights), S4 (weighted RGI), S5 (fast spindle frequency), S6 (SO–spindle coupling), S7 (REM continuity), S8 (1/f slope).

1. Relation to the MÖBIUS Equation

Main equation (manuscript):

$$P(M = \text{veridical} \mid S) = \sigma(\ \alpha \cdot \mathcal{P}(S) - \beta \cdot G(\theta, \gamma)\ )$$

Left-hand side. $P(M = \text{veridical} \mid S)$ is the probability that a REM sleep-generated sequence S is interpreted and stored as a veridical (autobiographical) memory M. Right-hand side. $\sigma(\cdot)$ is the logistic function. $\mathcal{P}(S)$ is a plausibility functional that maps the internal structure of S (mundanity, continuity, narrative coherence) to a scalar; higher values increase the odds of misbinding. $G(\theta, \gamma)$ is the REM sleep gating term that summarises oscillatory and neuromodulatory constraints, principally septo-hippocampal timing (θ), slow oscillations (SO)–spindle coordination, and cortical excitability (γ). α and β are scale parameters (see manuscript).

We formalise the REM sleep gating term as a normatively scaled composite of three neurophysiological pillars recorded from central electroencephalogram (EEG) (C3–C4): temporal coordination (SO–spindle coupling), REM sleep state stability (continuity), and cortical excitability (REM 1/f steepness). Larger values indicate tighter containment of dream content.

2. The REM sleep Gating Integrity (RGI) Index

RGI provides an operational proxy for $G(\theta, \gamma)$ using non-invasive EEG features recorded during NREM/REM sleep[2-4]. Higher RGI indicates tighter containment (stronger gating). The amplitude-free, central (C3–C4) definition uses three pillars of gating: temporal coordination, state stability, and excitability.

2.1 Feature vector (amplitude-free, central C3−C4):

$$x_i = (\ \text{logit}(r_i)\ ,\ C_i\ ,\ S_i\ )$$

where r_i is SO–spindle coupling vector strength (0–1) during NREM sleep; C_i is REM

sleep continuity (mean REM-bout duration, minutes); and $S_i = -$(aperiodic slope) is REM 1/f steepness (larger = steeper = lower excitability).

2.2 Control-only normalisation (normative z-scores):

$$z_{i,k} = (x_{i,k} - \mu^{ctrl}_k) / \sigma^{ctrl}_k , \quad k \in \{\text{logit}(r), C, S\}$$

The control means μ^{ctrl}_k and SDs σ^{ctrl}_k are estimated from the matched controls. All features are oriented so that larger z implies tighter gating.

Coupling is variance-stabilised with a logit transform; REM 1/f steepness is defined as the negative REM sleep aperiodic slope.

$$u_i = \log(r_i / (1 - r_i))$$

$$z_{i,\text{coord}} = (u_i - \mu^{ctrl}\_\text{logit}(r)) / \sigma^{ctrl}\_\text{logit}(r)$$

$$z_{i,\text{cont}} = (C_i - \mu^{ctrl}\_C) / \sigma^{ctrl}\_C$$

$$z_{i,\text{exc}} = (S_i - \mu^{ctrl}\_S) / \sigma^{ctrl}\_S$$

2.3 Composite forms:

Primary gating term (equal-weight composite)

$$G_{min}(i) = (z_{i,\text{coord}} + z_{i,\text{cont}} + z_{i,\text{exc}}) / 3$$

Minimal composite (primary):

$$RGI_{min}(i) = (z_{i,\text{logit}(r)} + z_{i,C} + z_{i,S}) / 3$$

Reliability-weighted gating term (sensitivity)

Weights are shrinkage-stabilised from per-feature effects (absolute Hedges' g, epic vs control).

$$w_k = |g_k| / (|g_k| + \tau), \quad \tau = \text{median}_k |g_k|, \quad \sum w_k = 1$$

$$G\_w(i) = \sum w_k \cdot z_{i,k}$$

Weighted composite (sensitivity):

$RGI\_w(i) = \Sigma_k w_k \cdot z_{i,k}$, with weights $w_k = |g_k| / (|g_k| + \tau)$, $\tau = \text{median}_k |g_k|$

where $|g_k|$ is the absolute Hedges' g (epic vs control) computed on the transformed, control-normalised scale. Weights are normalised so $\Sigma_k w_k = 1$.

2.4 Distance-to-control (sensitivity):

Signed distance-to-control (auxiliary index)

$$D_i = -\sqrt{(z_i - \mu_c)^T \Sigma_\lambda^{-1} (z_i - \mu_c)}, \quad \Sigma_\lambda = (1 - \lambda)\Sigma + \lambda I, \ \lambda \approx 0.2$$

Let z_i be the 3-vector of z-scores for subject i, μ_c the control centroid, and Σ the control covariance. With shrinkage covariance $\Sigma_\lambda = (1-\lambda)\Sigma + \lambda I$ ($\lambda \approx 0.2$), the signed distance is:

$$RGI_{DTC}(i) = -\sqrt{(z_i - \mu_c)^T \Sigma_\lambda^{-1} (z_i - \mu_c)}$$

More positive values indicate more control-like (tighter) gating. By definition $RGI_{DTC} \leq 0$ (control centroid = 0); "more control-like" means closer to zero (less negative). We report this signed form to keep "larger ⇒ tighter gating" semantics across all indices.

3. Concise Methods

3.1 Participants and Governance

We analysed eight adult PSGs: four clinically referred epic-dreamers investigated at the Sleep Disorders Centre, Guy's and St Thomas' NHS Foundation Trust (GSTT), London, and four matched controls drawn from the Canadian public repository MASS (O'Reilly, Gosselin et al., 2014), age/sex-matched as closely as possible. Controls were free of sleep pathology and had manual American Academy of Sleep Medicine (AASM) scoring (Berry, Brooks et al., 2017)[16]. All included subjects were ≥18 years of age and had no major psychiatric or neurological comorbidities, substance dependence, or use of medications known to alter sleep architecture. One epic case overlaps with a prospective case report focusing on persistent dream–reality confusion, from which phenomenological diaries were consulted for qualitative cross-validation[14].

Use of the clinical PSG data was approved under the Guy's and St Thomas' Electronic Research Records Interface (GERRI) framework, "Digital Biomarkers of Parasomnias" (IRAS 257283; REC 20/EM/0112), overseen by the Clinical Research Analytics Governance Group (CRAG). Analyses were conducted on fully anonymised retrospective

data within the GSTT secure environment, in compliance with the UK Data Protection Act and the General Data Protection Regulation (Regulation (EU) 2016/679). In line with the GERRI approval and national guidance for secondary use of de-identified clinical records, individual informed consent was not required. The study was carried out in accordance with the Declaration of Helsinki (WMA, 2013).

3.2 PSG and channel selection

Standard clinical vPSG montages were available (frontal F3/F4, central C3/C4, occipital O1/O2, EOG, EMG)[16]. For the present composite we used central C3–C4 as the primary site (fast spindle target) and treated frontal F3–F4 as a sensitivity analysis. Derived CSV exports provided spindle summaries, SO–spindle coupling tables, REM sleep fragmentation metrics, and REM sleep aperiodic slopes (2–30 Hz). No raw PSG was redistributed.

Channels and features: Primary derivation: central C3–C4 (fast-sigma target). Frontal F3–F4 was analysed for sensitivity. Features comprised SO–spindle coupling vector length r (NREM), REM continuity (mean REM-bout duration, minutes), and REM aperiodic slope (2–30 Hz), with steepness defined as −slope; fast spindle frequency is reported only in the weighted sensitivity composite.

Normalisation and statistics: Features were variance-stabilised (logit for r; steepness = −slope) and z-scored to the control distribution. Uncertainty is shown as bootstrap 95% BCa CIs (stratified by group, 20,000 resamples, BCa), and effect sizes as Hedges' g (small-sample corrected). Given $n = 4$ per group, inference is descriptive; emphasis is on direction and magnitude.

Control-only normalisation constants (C3–C4)

Feature	μ (control)	σ (control)
logit(r)	-2.244757	0.974119
REM continuity (min)	19.130208	6.031178
REM 1/f steepness (= −slope)	1.572947	0.070040
Fast spindle frequency (Hz)*	13.235960	0.086033

*used only in the 4-feature sensitivity composite.

3.3 Feature extraction (amplitude-free)

• Spindles: fast spindle frequency (Hz) from the C3–C4 row of the spindle summary (used in RGI_w only).
• SO–spindle coupling: vector length r from C3–C4 (radians converted only for internal checks); for composites we use logit(r).
• REM sleep continuity: mean REM sleep-bout duration (minutes) from hypnogram tables; larger implies greater REM sleep stability.
• REM sleep aperiodic slope: mean slope per REM sleep from C3–C4; we define steepness as −slope so larger implies lower excitability.

3.4 Normalisation and statistics

All features were variance-stabilised and normalised to controls (μ, σ). Group effect sizes are reported as Hedges′ g. Uncertainty for means uses bootstrap 95% BCa CIs (20,000 resamples). Given n=4 per group, all tests are treated as exploratory and emphasis is on effect sizes and directionality rather than null-hypothesis testing.

3.5 Data and ethics

Group-level summary tables and analysis code are described here in the Supplement; raw clinical PSG cannot be shared under audit ethics. Control PSGs were sourced from a public repository. Diaries for one epic case derive from a companion case report[14].

4. Distance-to-Control (Sensitivity)

We also compute a shrinkage Mahalanobis distance from the control centroid in z-space, using $\Sigma\_\lambda = (1-\lambda)\Sigma + \lambda I$ with $\lambda=0.2$, and report its signed form $RGI_{DTC} = -\sqrt{MD^2}$ so that larger values are more control-like.

Supplementary Table S2. Control-only normalisation parameters (C3–C4)

Feature	μ_ctrl	σ_ctrl
fast_freq	13.2360	0.0860
vec_logit	-2.2448	0.9741
rem_cont_min	19.1302	6.0312
steepness	1.5729	0.0700

Supplementary Table S3. Shrinkage weights and |g| (C3–C4)

Feature	Weight
SpindleFreq_fast	0.2037
SO_SpindleCoupling_logit	0.2132
REM_continuity_min	0.2987
REM_1f_steepness	0.2845

Supplementary Table S4. Composite group summaries and effect sizes (C3–C4)

composite	epic_mean	epic_sd	ctrl_mean	ctrl_sd	hedges_g
$RGI\_{weighted}$	-0.2017	0.7666	0.0	0.1862	-0.3144
RGI_{equal}	-0.1279	0.8489	0.0	0.1061	-0.1839
$RGI\_{minimal}$	-0.3187	0.2162	0.0	0.3261	-1.0017
RGI_{DTC}	-5.3959	3.757	-1.3915	0.1226	-1.31

Primary index: C3–C4 amplitude-free $RGI_{minimal}$ (coupling + continuity + 1/f).

C3–C4 Hedges' g (epic−control): $RGI\_{minimal}$ = -1.002; $RGI\_{weighted}$ = -0.314; RGI_{equal} = -0.184; RGI_{DTC} = -1.310.

Frontal sensitivity (F3–F4)

We applied the same control-only normalisation and transforms to frontal F3–F4 features and computed the minimal composite.

F3–F4 Hedges' g (epic−control): $RGI\_{minimal}$ = -0.706.

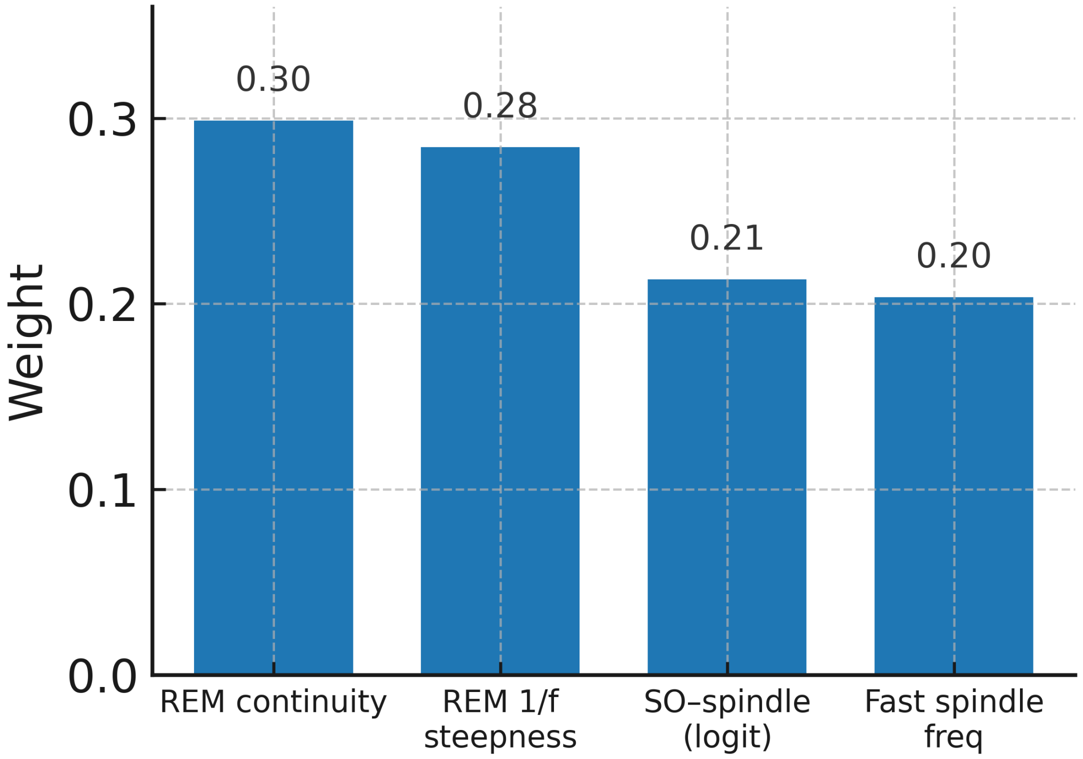

Supplementary Figure S3. Feature weights for the 4-feature RGI (C3–C4, amplitude-free).

Shrinkage weights $w_k = |g_k|/(|g_k| + \tau)$, normalised to sum to 1, where $|g_k|$ is the absolute Hedges' g for epic vs control on the variance-stabilised, control-normalised scale and τ is the median $|g_k|$. In this dataset, REM sleep continuity and 1/f steepness carry the largest weights, with SO–spindle coupling and fast spindle frequency contributing modestly.

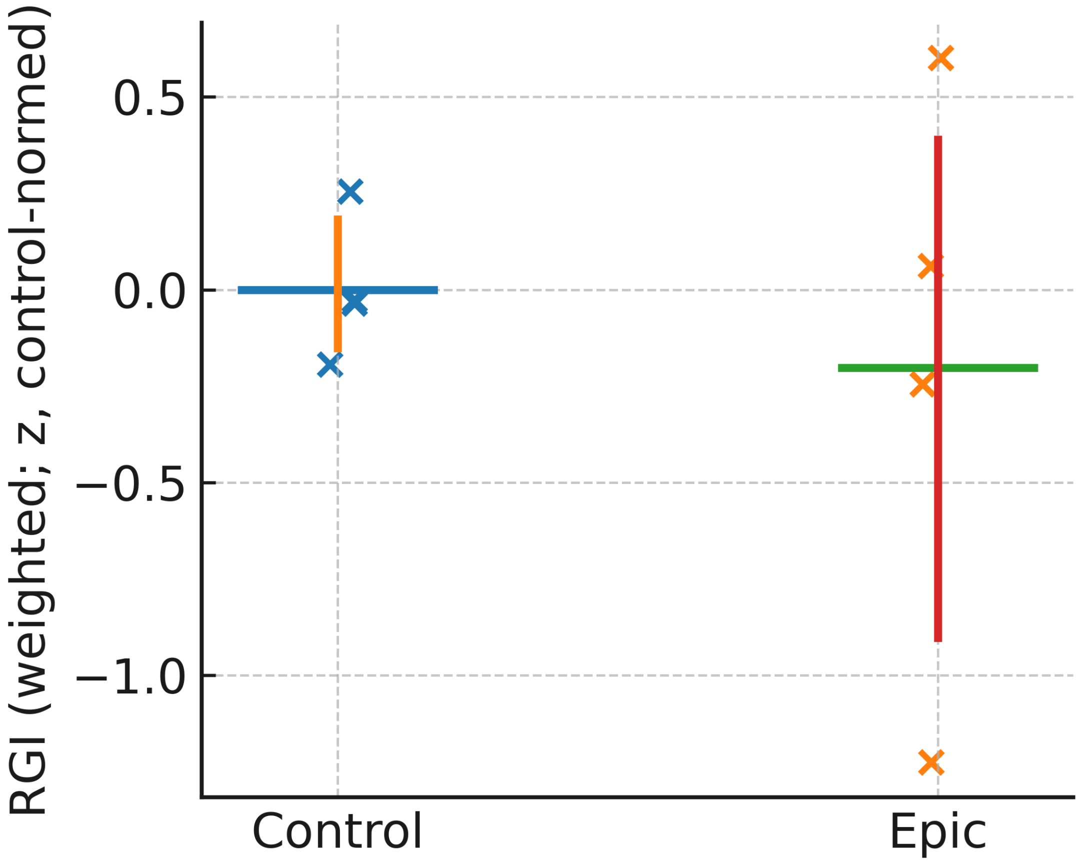

Supplementary Figure S4. Weighted RGI (4 features), central (C3–C4).

Same conventions as Supplementary Fig. S1 but using the shrinkage-weighted composite of fast spindle frequency, logit coupling, REM continuity, and 1/f steepness. Bars = means; whiskers = bootstrap 95% BCa CIs (20,000 resamples). Hedges' g (epic − control) = −0.314 (treated as a sensitivity analysis).

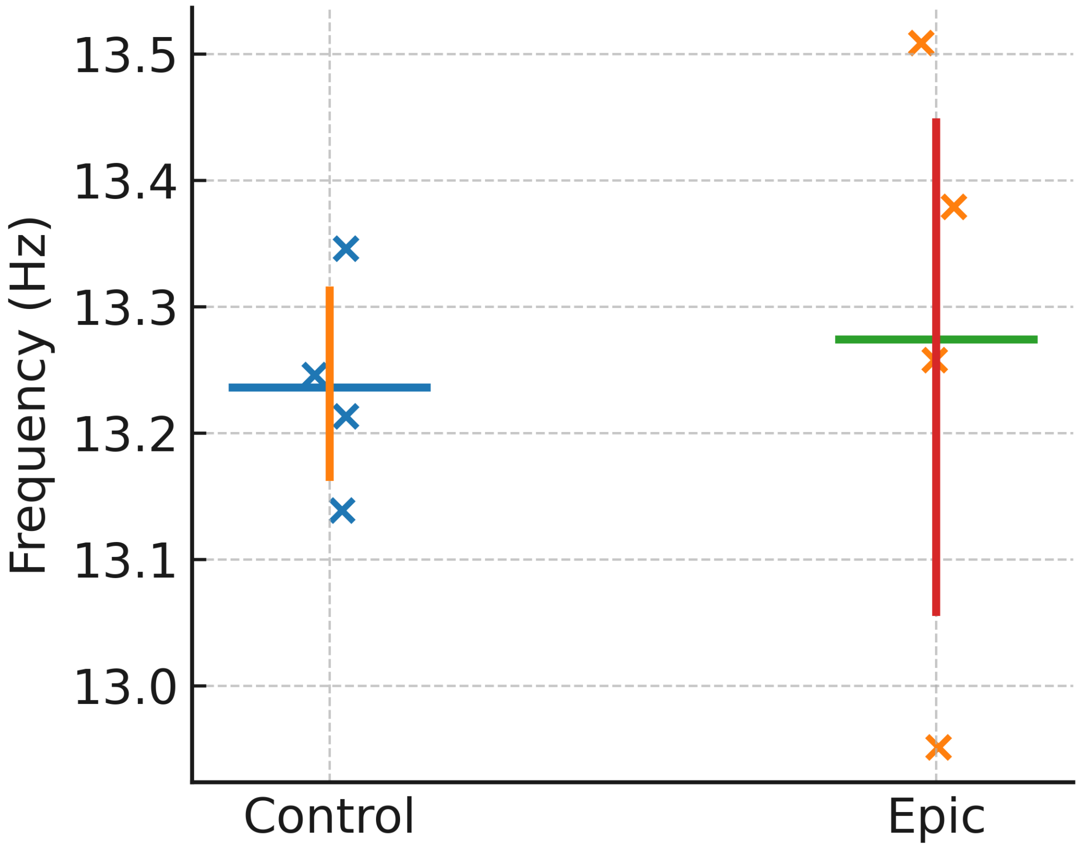

Supplementary Figure S5. Fast spindle frequency (C3–C4).

Values (points) with group means and bootstrap 95% BCa CIs. Spindles are the fast-sigma class; this panel documents the modest central frequency shift noted in the weighted composite.

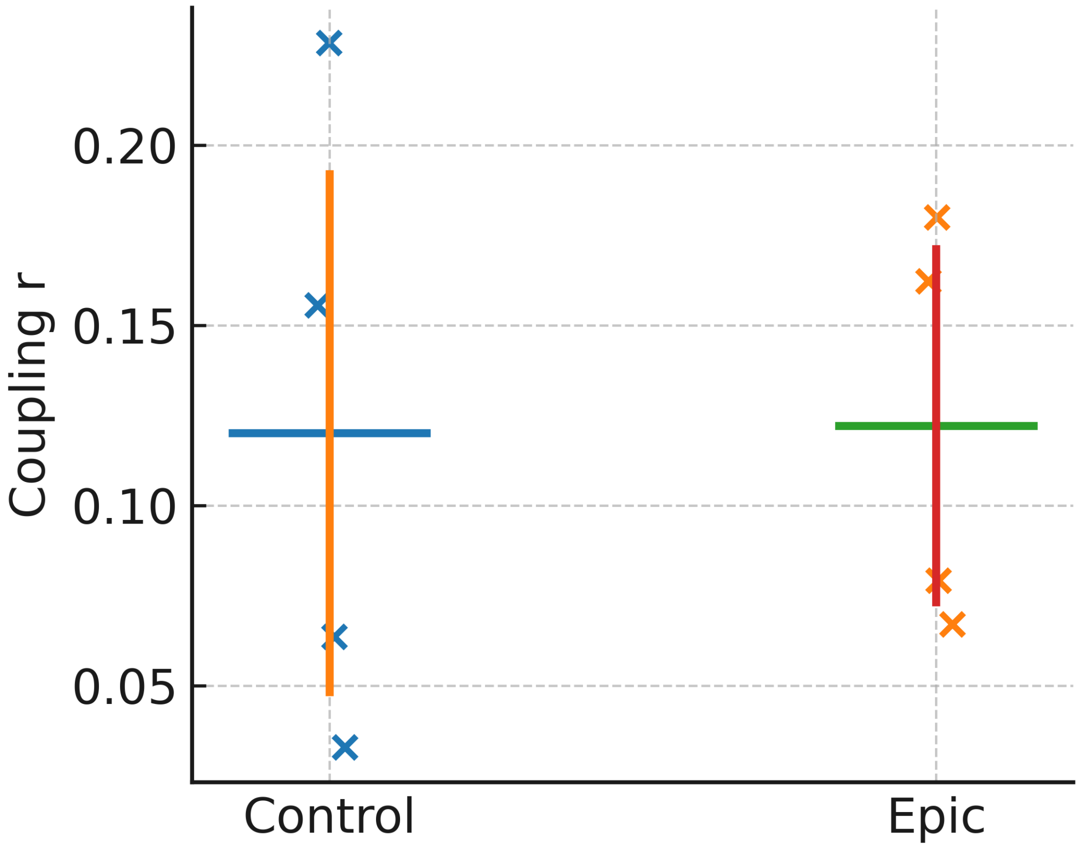

Supplementary Figure S6. SO–spindle coupling strength r (C3–C4).

Resultant length r ∈ [0,1] of SO phase at spindle centers; higher = tighter phase-locking. In the composite we use logit(r) prior to normalisation; this panel shows r on its native scale. Means and bootstrap 95% BCa CIs are overlaid.

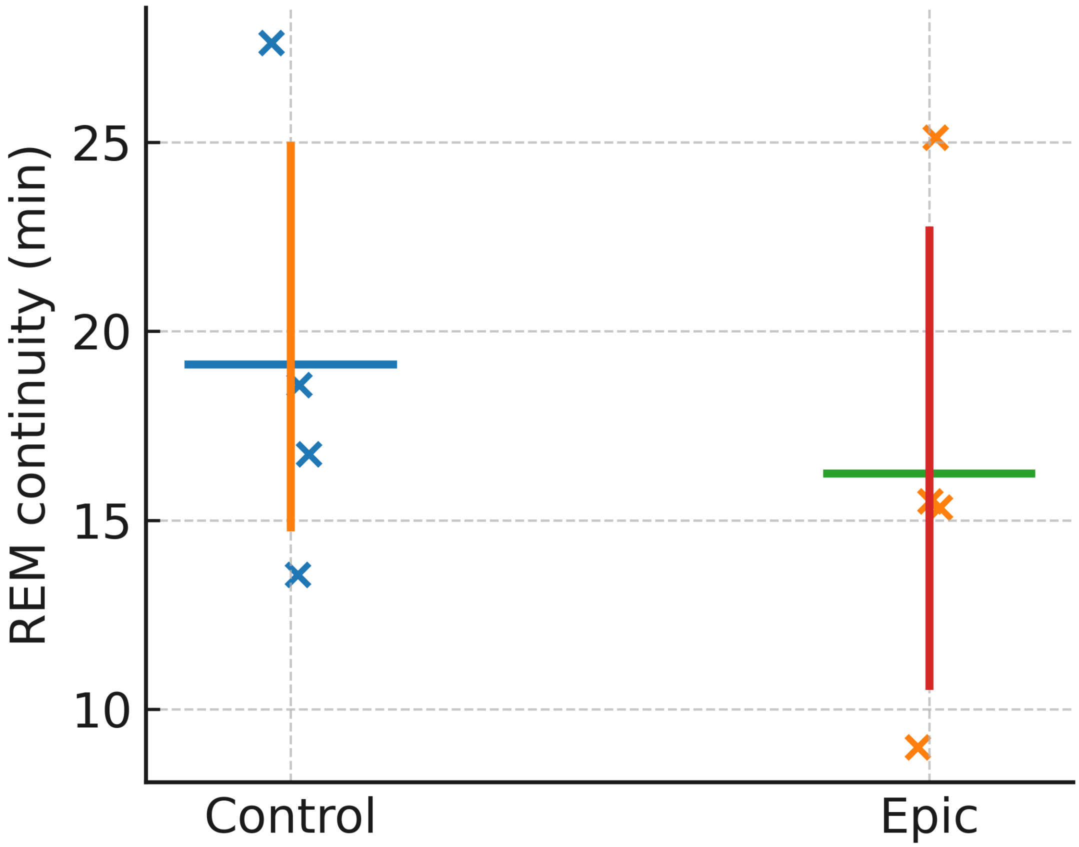

Supplementary Figure S7. REM sleep continuity (C3–C4).

Mean REM sleep-bout duration (minutes). Larger continuity supports intact REM sleep containment. This is the complement to fragmentation. Means and bootstrap 95% BCa CIs are shown.

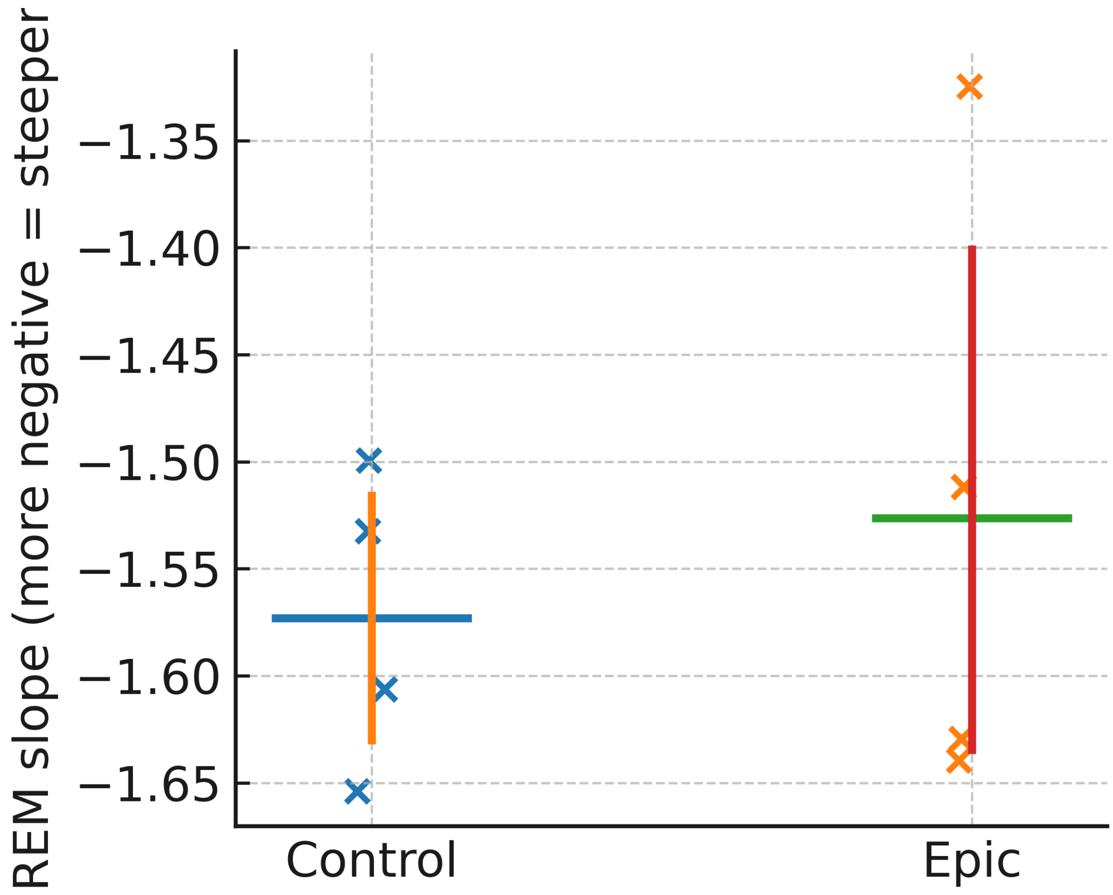

Supplementary Figure S8. REM sleep aperiodic slope (1/f), central (C3–C4).

REM sleep aperiodic slope fitted over ~2–30 Hz; more negative = steeper. In the composite we use steepness = −slope so that larger implies lower excitability. Means and bootstrap 95% BCa CIs are shown.

STAR Methods (one-page summary)

No in vivo animal work is reported, so ARRIVE guidelines do not apply.

RESOURCE AVAILABILITY – Data & code

Individual-level PSG recordings and clinical metadata for the epic-dreaming group are not publicly available owing to ethical and governance restrictions under the GERRI/CRAG framework at Guy's and St Thomas' NHS Foundation Trust. Group-level summary metrics underlying the plots are reported in the main text and Supplementary Tables. Control PSG data are available from the Montreal Archive of Sleep Studies (MASS[17]). Analysis scripts are archived on Zenodo (DOI: 10.5281/zenodo.18620608)[18]. No participant-level PSG data are included. Control PSG data are available from MASS (O'Reilly et al., 2014); access to the epic-dreaming cohort is governed as described.

EXPERIMENTAL MODEL AND SUBJECT DETAILS

Four epic-dreamers (Sleep Disorders Centre, London) and four matched controls (MASS[17]). One epic case overlaps with a Frontiers in Sleep report with dream diaries[14].

METHOD DETAILS

PSG montage included F3/F4, C3/C4, O1/O2, EOG, EMG. Primary derivation C3–C4; frontal F3–F4 for sensitivity analyses. Spindle metrics and slow-oscillation (SO)–spindle coupling were derived from standard detection outputs; REM sleep continuity from the scored hypnogram; aperiodic slope from REM sleep spectra over 2–30 Hz.

QUANTIFICATION AND STATISTICAL ANALYSIS

Coupling values were transformed with the logit; steepness = −slope. Control-only z-scaling used constants derived from the control group. The primary gate index G_min was defined as the equal-weight mean of the z-scored gating-related features (SO–spindle coupling, REM continuity, 1/f steepness). Sensitivity indices included Gw (a weighted composite with shrinkage weights) and a signed Mahalanobis distance with $\Sigma\lambda = (1-\lambda)\Sigma + \lambda I$ ($\lambda \approx 0.2$). We report means with bootstrap 95% BCa confidence intervals (20,000 stratified resamples) and Hedges' g for effects (Supplementary Tables S2–S4). In empirical analyses, we operationalised the theoretical gating term $G(\theta,\gamma)$ as RGI_min, the equal-weight composite of the three control-normalised features (see Supplementary Figure S2). Bootstraps are stratified by group; g uses the small-sample J correction and a small ridge term (10^{-12}) to avoid zero-variance resamples. Where p values are reported, they arise from exact randomisation tests enumerating all 70 label permutations for n = 4/4.

References

1. Parrino, L. & Rosenzweig, I. The futuristic manifolds of REM sleep. *Journal of sleep research* **34**, e14271 (2025).
2. Vandecasteele, M.*, et al.* Optogenetic activation of septal cholinergic neurons suppresses sharp wave ripples and enhances theta oscillations in the hippocampus. *Proceedings of the National Academy of Sciences of the United States of America* **111**, 13535-13540 (2014).
3. Zhang, Y.*, et al.* Cholinergic suppression of hippocampal sharp-wave ripples impairs working memory. *Proceedings of the National Academy of Sciences of the United States of America* **118** (2021).
4. Jarzebowski, P., Tang, C.S., Paulsen, O. & Hay, Y.A. Impaired spatial learning and suppression of sharp wave ripples by cholinergic activation at the goal location. *eLife* **10** (2021).
5. Izawa, S.*, et al.* REM sleep-active MCH neurons are involved in forgetting hippocampus-dependent memories. *Science (New York, N.Y.)* **365**, 1308-1313 (2019).
6. Kobayashi, Y.*, et al.* Properties of primary cilia in melanin-concentrating hormone receptor 1-bearing hippocampal neurons in vivo and in vitro. *Neurochemistry International* **142**, 104902 (2021).
7. Harris, J.J. & Burdakov, D. A role for MCH neuron firing in modulating hippocampal plasticity threshold. *Peptides* **172**, 171128 (2024).
8. Lu, Z.H.*, et al.* Melanin concentrating hormone induces hippocampal acetylcholine release via the medial septum in rats. *Peptides* **44**, 32-39 (2013).
9. Pimpinella, D.*, et al.* Septal cholinergic input to CA2 hippocampal region controls social novelty discrimination via nicotinic receptor-mediated disinhibition. *eLife* **10**, e65580 (2021).
10. Liu, J.-J., Tsien, R.W. & Pang, Z.P. Hypothalamic melanin-concentrating hormone regulates hippocampus-dorsolateral septum activity. *Nature Neuroscience* **25**, 61-71 (2022).
11. Huszár, R., Zhang, Y., Blockus, H. & Buzsáki, G. Preconfigured dynamics in the hippocampus are guided by embryonic birthdate and rate of neurogenesis. *Nature neuroscience* **25**, 1201-1212 (2022).
12. Mizuseki, K. & Buzsáki, G. Preconfigured, skewed distribution of firing rates in the hippocampus and entorhinal cortex. *Cell reports* **4**, 1010-1021 (2013).
13. Yang, W.*, et al.* Selection of experience for memory by hippocampal sharp wave ripples. *Science (New York, N.Y.)* **383**, 1478-1483 (2024).
14. Biabani, N.*, et al.* A non-neurodegenerative REM parasomnia with immersive dreaming and dream-reality confusion: a case report. *Front Sleep* **4**, 1659300 (2025).
15. Dragoi, G. & Tonegawa, S. Preplay of future place cell sequences by hippocampal cellular assemblies. *Nature* **469**, 397-401 (2011).
16. Berry, R.B.*, et al.* AASM Scoring Manual Updates for 2017 (Version 2.4). *Journal of clinical sleep medicine : JCSM : official publication of the American Academy of Sleep Medicine* **13**, 665-666 (2017).
17. O'Reilly, C., Gosselin, N., Carrier, J. & Nielsen, T. Montreal Archive of Sleep Studies: an open-access resource for instrument benchmarking and exploratory research. *Journal of sleep research* **23**, 628-635 (2014).

18. Rosenzweig, I. MÖBIUS REM-gating analysis code (COMMSBIO-25-8788A). (Zenodo, 2026).
